# The “Prudente Plan:” a São Paulo plan for the fight against cancer in Brazil, 1934-1954

**DOI:** 10.1590/S0104-59702024000100060en

**Published:** 2024-12-16

**Authors:** Elder Al Kondari Messora, André Mota

**Affiliations:** i Doctoral candidate, Department of Preventive Medicine/Faculty of Medicine/Universidade de São Paulo. São Paulo – SP – Brazil elderakm@hotmail.com; ii Professor, Department of Preventive Medicine/Faculty of Medicine/Universidade de São Paulo. São Paulo – SP – Brazil a.mota@fm.usp.br

**Keywords:** Cancer, Oncology, São Paulo, Philanthropy, History of diseases

## Abstract

This article investigates the strategies employed by Antônio Prudente to develop an initiative aimed at combating cancer throughout Brazil, based on his experience in the state of São Paulo. The approach used in the analysis draws on both the historiographical literature and primary sources, focusing on the period from 1934, when the São Paulo Association for the Fight against Cancer was founded, to 1954, when Prudente was appointed director of the National Cancer Service. The study highlights the disputes between cancerologists from the Union and São Paulo, highlighting the uniqueness of the São Paulo proposal, marked by a combination of liberal ideals and the sentiment of “Paulistaness.”

At the turn of the twentieth century, the state of São Paulo, Brazil, was held in great esteem by its leaders, being hailed as a “free society” with a “competitive economy,” whose “green gold” was a dynamo of progress and independence. This reference was to the prosperity of coffee farming, coffee having been Brazil’s main export product since 1840. Its expansion gave rise to a group with enough economic power to vie for political leadership in the country, including with representatives from the central power structure. Parallel to this it saw an upsurge in urban and demographic growth, boosted in part by immigration, which was associated with an exacerbation of health problems, as indicated in historical health records.

One of these pathologies was cancer, a mysterious disease that did not even appear in the São Paulo demographic yearbooks in the early 1900s, and was treated as one of a host of other minor ailments. However, over time, the number of deaths associated with malignant tumors increased significantly, making it a public health issue that could not be ignored.

The first organizations in the state dedicated to dealing with the problem emerged as of 1920, chief among which were the Arnaldo Vieira de Carvalho Institute and, in 1934, the São Paulo Association for the Combat of Cancer [Associação Paulista de Combate ao Câncer, APCC]. Cancer had been a subject of research in the northeastern state of Bahia since 1840, but this was associated primarily with interchanges with French science on the subject, not the number of cancer cases in the region. It was only in 1936 that the Bahian League Against Cancer was founded, revealing a lack of attention to this issue on the part of public authorities and society in general over a long period of time (Souza, 2014).

The Carlos Chagas Reform, undertaken in 1921, was crucial for shining a light on cancer. The new National Department of Public Health required all cases to be reported to the Inspectorate of Leprosy and Venereal Diseases, thus contributing to the progressive mapping of diagnoses. Other significant milestones at this time included the inauguration of the Rio de Janeiro Institute of Radiology, in 1919, and the Belo Horizonte Institute of Radium, on September 7, 1922 (Teixeira, 2010), followed a few years later by the Brazilian League Against Cancer and, in 1935, the first Brazilian conference on cancer.

The history of these initiatives shows how concern about the disease grew on a nationwide level, demonstrating the coherence and convergence of some initiatives in different states. However, the purpose here is to understand the peculiarities of this movement in São Paulo. To this end, this article is divided into three parts.

The first section presents the founding of the APCC, spearheaded by the surgeon Antônio Prudente, who took advantage of the public health infrastructure in São Paulo to address the imminent public health issue that cancer represented. The second covers the strategies Prudente and his wife used to develop a voluntary and philanthropic network to fight cancer, comparing them with what was seen in the Brazilian capital, Rio de Janeiro, and France. This project culminated in the construction of the A.C. Camargo Hospital, in 1953. Finally, the third part explains the intellectual and ideological foundations of the “Prudente Plan” and the political tensions this “national plan from São Paulo” to combat cancer sparked with renowned cancer specialists in Rio de Janeiro. It ends with the appointment of Antônio Prudente as director of the National Cancer Service, in 1954, where, although his term was short-lived, he nonetheless left a legacy.

Part of a broader research effort by Brazilian scholars to comprehend the history of the fight against cancer in Brazil, this study focuses primarily on the state of São Paulo over two decades, from 1934 to 1954. However, the analysis draws on information that extrapolates this timeframe, since we believe that historical events are interconnected by a “dialectic of durations” (Braudel, 1965, p.289) and that the understanding of certain facts depends on interactions with other medium- or long-term events.

The research draws on primary sources,^1^ which are contextualized within the historiography of cancer, whose chief contributors include Bodstein (1987), Bertolli Filho (2002), Teixeira and Fonseca (2007). For the content analysis, the concepts of representation (Chartier, 2002), philanthropy (Sanglard, 2010), *bandeirante* ^2^ medicine (Mota, 2005) are used, as well as the notions of permanent local network and specialized permanent vertical network (Merhy, 1992).

Using this methodological and theoretical approach, we were able to understand how the São Paulo proposal to combat cancer, promoted by the Prudentes, was intersected by broader historical contingencies, both national and international, and was intrinsically linked to liberal ideas and permeated by a strong feeling of “Paulistaness”^3^ (Ellis Júnior, 1934; Mota, 2005; Messora, Mota, 2018).

## Presages of war: Antônio Prudente and the Origins of the São Paulo Association for the Combat of Cancer

In the Spring of 1933, a series of articles on cancer appeared in *O Estado de S. Paulo* newspaper. Their author was Antônio Prudente Meirelles de Moraes (1906-1965), a medical doctor who had graduated from the São Paulo Faculty of Medicine in 1928. Shortly after earning his degree, Prudente set off for Germany to do an apprenticeship under Prof. Franz Keysser, a German physician who was known internationally for an electric scalpel he had developed to deal with previously inoperable tumors.

Many Brazilian physicians followed Keysser’s postulates, including Prudente, who, upon his return to Brazil, became an assistant in surgical techniques at the Faculty of Medicine and, in 1935, joined the Paulista School of Medicine as a full professor of reconstructive and plastic surgery. However, what assures this man, whose grandfather had been Brazil’s first civilian president, pride of place in this narrative was something else: his five articles in *O Estado de S. Paulo*, which were later collected in the book *O câncer precisa ser combatido* [*Cancer needs to be combatted*] (Prudente, 1935). These publications were a foretaste of his subsequent initiatives to fight the disease in São Paulo state, and presented Prudente as an authority on the subject.

His first text was on the global resurgence of research on malignant tumors, which he contrasted with the still incipient state of such research in Brazil. According to Prudente, this disparity was down to the apparent insolubility of the mysteries of cancer, caused by an “almost complete ignorance on the part of our people” (O problema..., 13 out. 1933, p.6). Nonetheless, he went on, cancer could be cured if it were diagnosed early enough. Therefore, a campaign was needed that had the power to overcome “stupid and often harmful preconceived ideas” on the subject (p.6).

Among such ideas were what Prudente called “cancerophobia,” which he claimed was spread by theories that sought to identify “cancer regionality” (Portugal, nov. 1910a, p.35) or “cancer streets or houses” (Ramos, set. 1911, p.163-164). Such theories were based on observations of concentric movements, starting from obituary evidence and funneling down to the smallest scale: “accursed houses” (Portugal, nov. 1910b, p.44; Ramos, set. 1911, p.163-164). The belief that such places were hubs from which cancer spread cannot be underestimated; indeed, so prevalent was it that it became a prerogative of the Inspectorate of Leprosy, Venereal Diseases, and Cancer.^4^

Since the nineteenth century, this perception had been reinforced by research published in São Paulo newspapers, most notably by Domingos José Freire Junior, who claimed he had discovered the “cancer microbe” (Messora, 2017, p.62). Although he subsequently fell into relative obscurity, his pioneering work against cancer was embedded in a perspective shared by a great many researchers at the time, namely, that the transmissibility of diseases had to be investigated experimentally. Pedro Severiano de Magalhães was one of those who shared such conceptions, publishing *Teoria parasitária do câncer* [*Parasitic theory of cancer*] in 1888. For his part, Freire developed a vaccine against malignant tumors, but it was not given to many people.

In the absence of an etiological definition, scientists from São Paulo recommended caution in view of the risk of contagion. Olympio Portugal (nov. 1910a, nov. 1910b), a member of the São Paulo Society of Medicine and Surgery, was one of the voices that reinforced this perception, comparing cancer to tuberculosis. In this context, it was not long before “accursed houses” became the targets of prophylactic measures by the General Disinfection Service, even in the absence of any definitive scientific evidence. Such measures can be seen, rather, as “playing it safe” on the part of the authorities (Araújo Neto, 2019).

The impact of this representation extended into the 1950s, in association with the miasma theory, such that it was common for people to cross to the other side of the street when they were approaching A.C. Camargo Hospital. Another indication of this phenomenon is the fact that during its construction, there were complaints about installing such a hospital in a populated area of the city. At these times, Prudente would go to the newspapers and reinforce his message: “One of the absurdities that is spread is that cancer is a contagious disease, and that cancer patients should not stay in such a central hospital. Cancer is not contagious, according to the conclusions of all the International Congresses held to date. Anyone who may consider it thus should, in my opinion, demonstrate it immediately for the good of all humanity” (Notícias..., 27 jun. 1944, p.5).

Prudente demonstrated a certain alignment with the embryonic theory of cancer. In his second article, he argued that tumors originated from malformations or mutations of tissue during its development phase, for which reason he required his patients to pay attention to scars, warts, cavities, and papillomas. This perspective gained strength as the prevalence of other diseases, such as yellow fever, tuberculosis, and trachoma, diminished in response to public health interventions, while cancer grew vertiginously.

Prudente’s third article was dedicated to cancer statistics, number of deaths, and the ethnographic distribution of the disease. This last issue had to do with the “degree of civilization of different peoples” (O problema..., 9 nov. 1933, p.4). He was not the only physician to hold that “cancer is more common in civilized countries than [illegible] of primitive peoples” (p.4) and that the disease was far more common in women than in men. At the end of the text, he introduced readers to the category of “occupational cancer,” which he explained was a disease related to work activities.

In the fourth and fifth articles, Prudente (1935) explained how cancer evolved, the causes of death, the symptoms, how to prevent it, and other issues, all of which essentially converged in the same direction: the need for an organization to fight cancer in São Paulo.

In fact, historical contingencies would favor Prudente’s aspirations. In the 1890s, a new republican order had emerged, under which public health issues had been transferred to the state level, prompting São Paulo to reformulate its organizational structure. In the field of health, this had been materialized in the development of the first Sanitation Code, in 1894, and the organization of the Sanitation Service, between 1891 and 1892. This service was subordinated to the Secretariat of the Interior and had several bodies, such as the Directorate of the Public Health Service, the Isolation Hospital, the Mental Asylum, the General Disinfection Service, laboratories for chemical, bromatological, and pharmaceutical analyses, an institute of bacteriology, an institute for vaccine production, and the Public Health and Demographics Statistics Section. Shortly afterwards, the Butantã Institute was founded in the west of São Paulo, focused on research into mental illnesses (Mota, 2005; Escorel, Teixeira, 2008).

According to the literature, no other state had such a comprehensive public health apparatus; indeed, its cost consumed a significant proportion of the state budget (Ferreira, Luca, 2011). However, this was the way that its leaders proposed to deal with health problems, especially the devastating rates of malaria and yellow fever in Santos and Campinas and the smallpox in São Paulo city. There were also cases of bubonic plague, cholera, and gastrointestinal infections, all of which were connected in some way to the urban expansion and socioeconomic development in the region (Ribeiro, 1993).

The contagious nature of these diseases meant an authority was needed that had the capacity to oversee the whole state and its population, even if the measures it took might curb certain individual freedoms or violate political autonomy and even property rights, especially when the patients – most of whom were classified as poor – were deemed a collective threat due to the potential of their ailments to be transmitted to the wealthy classes (Hochman, 1998).

The São Paulo health apparatus at Prudente’s time had been created in response to this and the difficulties it posed, constituting the context in which cancer appeared on the public health agenda and helping us understand Prudente’s own endeavors. As São Paulo city took a stance as a bastion of modernizing actions, in line with the scientific achievements of the period, it is no surprise that the “*bandeirante* medicine” (Mota, 2005) decided to tackle what had been dubbed the “disease of great civilizations.”

The first hospital for cancer patients was built in the 1920s on the initiative of Arnaldo Vieira de Carvalho. Leadership in promoting the cause was subsequently taken over by Antônio Prudente, who seized it as a key moment for consolidating the ideology he felt should underpin future actions, in view of the deficiency of state action in the 1930s (Teixeira, Fonseca, 2007). As he put it, the “cancer problem in our land can only be resolved within the living conditions of each federative unit^5^ and in accordance with the material and intellectual possibilities of these same units” (Prudente, 1935, p.168).

Prudente argued that public bodies should take urgent action to tackle the issue and that an Inspectorate for the Combat of Cancer should be created along the same lines as the existing inspectorate for leprosy. However, as public policies were geared primarily towards dealing with rural endemics and epidemics that threatened the population, “alas,” he lamented, “creating this new Public Health Service department is not considered” (Prudente, 1935, p.172). All that remained was for the private sector to take up the fight against malignant tumors, and it was based on this understanding that Prudente made this mission his own. Accordingly, the APCC was founded as a philanthropic entity in 1934, representing, in his view “the needs of the State in the cancer problem” (p.176).

By this time, there was a medical consensus around the need to prioritize early diagnosis for cancer treatment. The APCC therefore took responsibility for two problems that hindered prophylaxis in the state: “The complete ignorance, on the part of laypersons, of data relating to the disease, and the deficit of knowledge about cancer, on the part of doctors, dentists, pharmacists, midwives, nurses etc.” (Prudente, 1935, p.171). To meet this demand, an effective “anti-cancer” propaganda campaign was rolled out, using media ranging from posters, pamphlets, and newspapers, to cinematographs and radio, in a bid to improve knowledge on the subject and thus facilitate early discovery of the disease.

While these actions in São Paulo were matched by those of other leagues elsewhere in the country, there were three aspects of Antônio Prudente’s initiative that made it unique. The first concerns the strategies and rhetoric he and his wife, Carmen Prudente, employed, taking advantage of the representations of cancer and a wide network of influential relationships. The second was a corollary of the first: by the mid-1930s, a philanthropic association had taken shape that coordinated and galvanized actions and care for cancer, formalizing the fight by the people of São Paulo state against the disease by means of an approach that used private means to address a “public health policy.” Finally, the third has to do with the favorable circumstances in which the couple executed their strategies, galvanizing public opinion as well as the French experience on which they were based.

## Philanthropy and Paulistaness: strategies employed by the Prudentes and the founding of A.C. Camargo Hospital

In the late 1800s, there was a growing perception in the medical community that a range of problems, especially those linked to poverty, could not be left at the mercy of the charitable initiatives of a small portion of the population. Under French influence, Brazilian physicians began to defend the need for forms of public healthcare to be organized in the country, to be planned and executed by the government through an institutional structure, with skilled personnel and efficient, effective measures, as dictated by the prevailing scientific knowledge (Martins, 2011).

Antônio Prudente, acting under this very influence, stated:

We could do something similar, especially in the State of S. Paulo, where the environment could already support such an organization. In fact, our Health Service is so well equipped that it would not be hard to achieve a perfect organization for the entire State, even taking advantage of its materials and employees, not only from the point of view of research and treatment, but also with regard to anti-cancer propaganda.

... I am sure that with the help of the population itself, and as part of the State Health Service, we would soon be on a par with the most advanced countries (O problema..., 7 jan. 1934, p.6).

The public authorities were not, however, persuaded by his arguments, which is why he ended up founding the APCC. However, this association only started to make real ground in the 1940s, when it received a donation of land from the São Paulo state government. The donation was formalized in the presence of the federally instated interim governor, Fernando Costa, and the director of the Buenos Aires Cancer Institute, Angel Roffo, who was not only a renowned scientist in his own right, but had worked with his wife, Helena Larroque, to create a civil association to organize the fight against cancer in Argentina, in 1921 (Buschini, 2014).

The plans for São Paulo included the construction of the Central Cancer Institute, later renamed the Antônio Candido de Camargo Hospital – an organization that epitomized the links between the moral conceptions of Christian charity and philanthropy, constituting a public, rational model of a kind that gained ground throughout Brazil in the early decades of the twentieth century (Sanglard, 2010). This was enhanced by the endeavor to galvanize the latest advances in medicine, conceptions of cancer, public health organizations, and the material possibilities for their expansion. It was this tangled web of influences that explains the engagement of wealthy women and men with the realm of healthcare, epitomized in the case of the fight against cancer in São Paulo by the figures of Antônio and Carmen Prudente.

If Antônio Prudente was able to galvanize important philanthropists around his cause, such as Commander Martinelli, who donated five million cruzeiros to APCC (Martinelli, 29 dez. 1943), Carmen Prudente went further. In 1946, she founded the Women’s Network for the Fight against Cancer, which held fundraisers of various kinds and “maintained a register containing the telephone numbers of much of the São Paulo elite, whose members were invited to make monthly contributions to the campaign for a hospital in the city. Membership of the entity was such that it managed to attract more than 25,000 women to its ranks” (Teixeira, Porto, Noronha, 2012, p.51). It was a pioneering initiative that sparked the creation of several other women’s leagues around the country, mainly as of the 1950s.

This was a time when women’s participation in public life was limited, because bourgeois sensibilities, prevalent since the early 1900s, dictated a strong ideological separation between domesticity and sentiment, on the one hand, and public life and rationality, on the other. It was this exclusion of women from the public sphere that meant philanthropy ended up being a primarily female activity, insofar as women (or so believed their contemporaries) were better suited to do-gooding and caring for those in need than men, while also having plenty of free time to organize charitable associations and raise funds for good works (Martins, 2015). It was in this context that Carmen Prudente’s philanthropic work provided a crucial intersection between the public and private spheres.

It was between 1930 and 1945, during the Estado Novo, an authoritarian regime led by Getúlio Vargas, that ladies with means felt empowered to intervene socially, which meant the work carried out by Carmen Prudente and other women was not perceived as a breach of morals or virtue; on the contrary, it was a channel for “female citizenship,” based on women’s essential social utility and vocation: motherhood (Martins, 2011). Interestingly, the idea that caring was a natural facet of motherhood was extended even to childless women. The Prudentes were a case in point: “We had no children,” Carmen used to say, “except for one, made of concrete,” referring to the hospital (Bueno, 2015).

The Prudentes’ action was successful, not least because it took advantage of a phenomenon of an overwhelmingly symbolic nature: “Paulistaness.” This was a sentiment that translated into a constructed narrative that was widely disseminated at the time: that “the *bandeirantes* saw no harm in their reckless endeavors” and would overcome “all obstacles” (Ellis Júnior, 1926, p.353). It is no coincidence that the couple’s rhetorical strategy was so rich in praise for the people of São Paulo, their patriotic resolve and selfless collaboration, especially as “the state and federal governments have contributed so little to such a great work, as they have not given even 10% of the total donated by the population of São Paulo, which has topped 30 million cruzeiros” (Initiada..., 22 maio 1952, p.11).

These numbers were the result of several fundraising campaigns, such as the Brick and Cement Campaign and the Working Day campaign. The idea behind this latter campaign was that factory employees should donate a day’s wages to the hospital. Other events included “anti-cancer” exhibitions, which raised funds via admittance fees, as well as balls, movie tickets, and even soccer matches. The members of the Women’s Network had their own campaign, which they called the Crab Race: a golden crab, donated by a well-known jewelry company, was awarded to whomever managed to collect the largest amount in donations (Nenhum..., 18 maio 1948, p.2).

A group was also set up within the APCC, called Commanders against Cancer, aimed at privileged investors, such as industrialists, business leaders, and other high-net-worth individuals. Its members were awarded army ranks according to the amount they donated, with this rank being updated at each monthly Commander meeting (Campanha..., 19 jun. 1952, p.11). In parallel to the “military” event was the Magic Towel expedition, in which a long piece of fabric was divided into 32 parts, which were distributed among the wives of the “commanders” and “officers.” These women then had to get people to sign their towels, in exchange for a thousand-cruzeiro donation, with a maximum of 30 signatures per towel. This strategy was so successful that the Women’s Network replicated it at more affordable prices (Campanha..., 28 maio 1952, p.11).

These were some of the measures designed to combat a disease that, despite being conceived as a public health problem, garnered relatively little action on the part of the authorities. The appeal to São Paulo patriotism was one of the strategies that nurtured a certain popular determination in this fight. It is also worth mentioning the power of the representations or metaphors for cancer (Chartier, 2002; Sontag, 1984), the fear it caused, and the credibility the philanthropic activities acquired.

Unlike charity, philanthropy did not bear the virtue of piety; it was a secular activity of social utility. For it to work, people had to be brought together who “participate in the same movement of expression and identity of donor: they relate to the convictions, they place it in a social space, and inscribe it within a group of relationships” (Sanglard, 2010, p.128). This is what the Prudentes did, mobilizing groups of people for their cause and taking action where the government failed to. This experience was different from what was seen in the capital, Rio de Janeiro, but similar to that of France, where women took the helm in promoting the construction of hospitals.^6^

Nonetheless, the solidarity of their actions does not mean there were no other motivations at play, such as a desire to garner academic and professional prestige (amid the creation of a specialty such as cancerology) and the ambition to be a leading light of a cause with national and international scope.

In fact, São Paulo was not the first place in Brazil where philanthropy and cancer institutions crossed paths: a sizeable portion of the philanthropists in Rio de Janeiro still bore the titles of the erstwhile imperial nobility: barons, counts, viscounts, baronesses, and viscountesses. However, it was a well-known industrialist, Guilherme Guinle, who spearheaded the construction of a cancer hospital there. In France things were not so very different, where contributions to combat the disease came from both the nobility left over from the *Ancien Régime* along with politicians and industrialists.

In Rio de Janeiro, Guinle’s actions were not covered in the press or even in specialized periodicals. Accordingly, the first plans for the cancer hospital, designed and presented in 1925, never got off the drawing board. Ten years later, with donations from a few private individuals and still no federal government funding, Guinle himself was on the verge of giving up on this project (Sanglard, 2010). It was only when Getúlio Vargas came to power, centralizing state actions, that the Cancer Institute became a reality.

In France, the origins of the fight against cancer dated back to the context of the First World War. The patriotism that bloomed in the midst of military hostilities was favorable for charitable donations to the French health system, as the war effort meant all men were expected to serve in the army, whether they were young or old. The more advanced age of these older combatants indicated that the incidence of cancer among soldiers was not so very rare, making it a cause for concern of the country’s military medical authorities (Pinell, 2004).

This situation served to make the French aware of the social dimensions of cancer. Soon, when public health authorities decided to act on the issue, they adopted the strategy of drawing on well-known personalities, who ranged from former nobility to common citizens, all in solidarity with the servicemen. In 1918, the Franco-Anglo-American Anti-Cancer League was set up, chaired by French politician Justin Godart, with the consent of the British and American ambassadors (Pinell, 2004).

It would not be a stretch to draw a parallel between the patriotic call to action by the French and the appeal to the “populations of São Paulo” to join the fight against cancer, in view of the fact that in both cases the act of stirring up specific emotions was instrumental in securing the goodwill of the people. So it was that the Central Cancer Institute (A.C. Camargo Hospital) was founded in 1953, the result of an alliance between medical doctors, philanthropic organizations, and the people of São Paulo. Proud, Prudente saw the institute as “a desire of our people, an aspiration of many who contributed to its realization” (Inaugurado..., 25 abr. 1953, p.11). In contrast, the movement in Rio de Janeiro did not have the same impact or broad public support, forcing the government to make significant interventions in the construction of the infrastructure needed to fight the disease.

If this were the end of the story, it would already be a great achievement, but Antônio Prudente had other ambitions: he wanted his project to extend throughout the country. He chose to submit his proposal to the federal government at a time when the director of the National Cancer Service was out of the country, causing friction with oncologists who worked in Rio de Janeiro.

## The “Prudente Plan:” a São Paulo plan for the fight against cancer across Brazil

The 1940s was a period of centralization under the Getúlio Vargas regime, which had an impact on public health. Thirteen national services for infrastructure and the control of specific diseases deemed to be a priority at the time were created. These included the National Cancer Service, created through law no. 3643, of September 23, 1941, which was part of the National Department of Health, itself part of the Ministry of Education and Health.

Germane to our discussion is the fact that the National Cancer Service ensured a definitive place for cancer on the public health agenda for the entire country (Brasil, 23 set. 1941). Its first director was Mário Kroeff, a physician from the southernmost state of Rio Grande do Sul. After appraising Vargas as to the problem of cancer, he managed to get him to pass an executive order (no. 378, of January 13, 1937) to create the Cancerology Center – the embryo of the National Cancer Service – to which he was soon appointed director. Kroeff was unable to begin the activities that he considered central at that time, but he took it upon himself to learn about the best practices and appropriate the most advanced devices for cancer treatment, traveling to the United States in 1942 for this purpose.

Parallel to this, the status of the APCC had risen to the point that it even rivaled the group of cancerologists in Rio de Janeiro. Prudente sent letters to Vargas in which he stated that the Union’s expenses would not be so high, being incurred only for the maintenance of treatment units. He backed up his claim by reporting that he would ask a renowned patrician from São Paulo for direct donations, and that the president could even use this fact as political leverage (Prudente, 31 jan. 1943) – a request that resulted in the donation by Martinelli mentioned above.

It was no coincidence that Prudente reached out to the highest echelons of the federal government precisely when Kroeff was in foreign lands. He sent the Minister of Education and Health a project entitled the National Network Against Cancer, saying that “the need for this Network is imposed by the almost total neglect of cancer patients” (Prudente, 31 jan. 1943).

The most striking feature of the plan was the decentralized organization it proposed, which made it ideologically liberal. Prudente divided the country into several regions, each of which should receive cancer treatment centers. He was opposed to the National Cancer Service model, as he considered it “a utopia to organize an excessively centralized fight against cancer” due to the discrepancies in the population distribution of the country. Rather, the disease should be fought by a network covering every region, established by different units (Prudente, n.d., p.2).

At this point, we should stop and look back at the historical contingencies that sustained and nourished Prudente’s proposal.

Since its origins, in 1892 (with the founding of the Sanitary Service), São Paulo’s public health infrastructure had been organized and provided by municipal and state governments, with the sponsorship of coffee growers, with the Union exerting little direct influence on its provisions.

From the mid-1920s onwards, the health policies that took effect were inspired by the American model of health centers, promoted by the Rockefeller Foundation and the Johns Hopkins School of Hygiene and Public Health, where the then director of the Sanitation Service, Geraldo Horácio de Paula Souza, had studied (Campos, 2002). Under his administration, which lasted from 1922 to 1927, health centers were set up throughout São Paulo city, and health educators were responsible for teaching the population the basics of good hygiene. The ideas behind this model, called the “permanent local network,” were similar to the liberal-democratic projects that lost favor as of the 1930s, under President Vargas. The replacement was a different kind of service, called a “specialized permanent vertical” service (Merhy, 1992, p.27).

The “permanent local network” model presupposed a relationship between state action and social sectors through health services, based on an organization capable of dealing with collective health problems, which could be mitigated or remedied through the health awareness of each subject. In contrast, the second model was structured by public bodies that acted on specific public health problems, controlling them on an individual and collective level through specialized care.

Prudente was not far from the second model, if we consider that oncology required costly technologies and the specialization of human resources. It is no coincidence that in the 1930s, at the same time that “specialized permanent vertical” services (Merhy, 1992, p.27) were implemented, Prudente himself began publishing articles in the press and founded the APCC. However, public health in São Paulo at this time was still based on the Sanitation Code, which was amended to allow for health centers, but was influenced little by the Union, even under Vargas, which is explained by the high turnover of directors in the Health Service (Mascarenhas, 1949).

Given these conditions, we understand that Prudente’s proposed network actually drew on both models. He accepted that sophisticated specialization in cancerology was needed, which implied the verticalization of care, but this would be adapted to allow for a distributed network of hospital and health education facilities, as would be consistent with a “permanent local network” (Merhy, 1992, p.27).

The liberal nature of the proposal was materialized in the call for an autonomous network distributed in units, rather than a strong and centralized organization. This conception influenced how the network would be financed, given that the government would have to spend less, leaving the individual taxpayer with an optional role. This was one of the arguments Prudente used to win Vargas’s support for his plan: he recommended obtaining the funds needed to execute his project by appealing to the generosity of the people, proposing a National Campaign against Cancer under one of the president’s own catchphrases: “We must, first of all, alarm the people!” (Prudente, n.d., p.13).

Prudente’s next step was to advocate an organization exactly like the APCC and the Women’s Network for the Fight against Cancer, established by Carmen Prudente, but with a nationwide footprint. This would mean the National Cancer Service was not the only central organization:

In each city that is the headquarters of a Unit from the Network, two committees must be organized. The first will be made up of physicians and local representatives, under the Campaign’s local Executive Committee. The second will be made up of ladies only, with the task of requesting and collecting donations (Prudente, n.d., p.13).

Finally, Prudente offered to take on the position of “technical director” of the campaign, with responsibilities that would cover the entire country. This proposal was particularly attractive for Vargas, since the Ministry of Education and Health was focused on prevalent diseases, such as yellow fever and malaria (Teixeira, Fonseca, 2007, p.78). Therefore, the idea of private institutions dealing with cancer, with modest federal and state funding, seemed more appealing. Without delay, he gave the green light for the National Campaign.

Nonetheless, there was already another “campaign against cancer” underway, which was announced precisely with the creation of the National Cancer Service. This campaign was similar in every way except the weight attributed to the population’s own voluntary and contributory actions. According to the founding document of the institution, the National Cancer Service was responsible for organizing, orienting, and overseeing a nationwide campaign that would involve investigating cancer, gathering epidemiological data, taking prophylactic measures, and treating patients, as well as monitoring their progress after treatment or providing institutional care for those most in need (Bodstein, 1987). However, it makes no mention of fostering fundraising from the population by raising awareness about the disease. This was not to say that the institution did not receive patronage from individuals or that it did not count on the work of voluntary groups; but these activities were not formally prescribed, nor were they a direct objective or a regular occurrence.

This type of contribution gained some prominence in the government body, mainly as of 1948, because Kroeff wanted to “have an impact on the public, who, in his view, were oblivious to the cancer issue” (Teixeira, Fonseca, 2007, p.98). To this end, a partnership was established with the Jockey Club with the purpose of devising the National Cancer Service’s first educational campaign about cancer. Three years later, new funds and a stronger institutional presence were achieved when Napoleão Laureano, a physician and local councilor from northeastern Brazil who was suffering from terminal cancer, coordinated a campaign to create a cancer hospital in his region of origin, which yielded a significant number of donations for the Laureano Hospital foundation, in addition to providing evidence for Kroeff. These examples show how the director of the National Cancer Service followed the lead of Antônio and Carmen Prudente.

The approach Kroeff took could be seen as a reaction to Prudente’s proposal. In other words, the São Paulo project for Brazil did not pass him by, even when he was on his scientific exchange in the United States.

It was Sérgio de Barros Azevedo, the interim director of the National Cancer Service, who received the news about the “Prudente Plan.” Upon its receipt, he wrote to the government authorities that Prudente was “naturally not fully aware of our program and our activities, which continue to be carried out intensively and with the greatest sacrifices.” After all, his proposal “in general terms coincides with the program that is already established” (Azevedo, 3 mar. 1943). However, as this program had not yet been submitted to Minister Capanema, Azevedo had to quickly set the record straight. The following day, he wrote another letter in which he explained that the program was already ready, but that it had not been delivered because he was waiting for final approval by Kroeff, who was away at that time. He added that the project took care of “financing in a more practical way than that suggested by Dr. Antônio Prudente” (Azevedo, 4 mar. 1943).

Upon learning of the situation, Kroeff wrote a detailed letter to Capanema, which is filled with discouragement and disappointment. He criticizes Prudente’s arguments, which were submitted in his absence from the country, for clashing with the interests of the National Cancer Service itself:

Now, however, the news coming from Brazil by telephone about your plans bring me personally the disillusionment of men and the conviction that our endeavors for the public good are precarious when they depend on others. I must confess that I consider the reform of the National Cancer Service, undertaken in my absence, to be disrespectful to its director and an act of ingratitude to myself, given the services I have provided to patients and to my country through medicine ( Kroeff, 9 mar. 1943).

It is curious that Instituto Arnaldo Vieira de Carvalho and the Humberto Primo Hospital, both in São Paulo, were regarded as “branches” of the National Cancer Service, but not the APCC. It was only after Kroeff’s return – and to relieve the ill-feeling that had been created –that it was incorporated (Brazil, 19 out. 1943), which we interpret as the direct result of Kroeff’s efforts.

For the APCC to be officially affiliated, it had to submit its bylaws for approval by the president of the Republic – a process in which Kroeff intervened. In correspondence with Minister Capanema, “thinking, however, it should be in an official capacity” (Kroeff, n.d.), he suggested a few amendments:

In article 1 of the São Paulo Association for the Combat of Cancer, where it reads ‘with action throughout the national territory,’ include ‘subordinated to the National Cancer Service.’

In article 4, letter b, where it reads ‘Cancer Institute,’ put ‘São Paulo Institute of Cancer’ or ‘São Paulo Regional Institute.’

... this title Cancer Institute could in the future cause confusions of nomenclature and duplicated powers, as the National Cancer Service is responsible for executing and controlling the fight against cancer throughout the national territory (Kroeff, n.d.).

This circumstance leads us to conclude that unlike in France, where rivalry between surgeons and radiologists caused disputes over the conception and treatment of cancer (Pinell, 2004), in São Paulo and Rio de Janeiro, tensions between physicians were related to the ideological principles underpinning their projects and consequently the question of who should lead the fight against cancer in the country.

The conclusion of the previous paragraph corroborates what Andrade and Lana (2010) found in a study of the institutionalization of the fight against cancer in Brazil: that there was a dispute for primacy and protagonism in the propositions for coordinated actions in this respect. However, the same authors’ comparison of the Prudente Plan and the National Cancer Service’s plans (Table 1) concluded that the respective leaders shared similar assumptions about the structuring of public health policies for cancer. Nevertheless, these similarities hide the ideological differences that fueled their respective proposals.

**Table 1:** : Proposals for the National Network against Cancer and the National Plan for the Fight against Cancer

National Network Against Cancer	National Plan for the Fight Against Cancer
Construction of a national cancer institute	Construction of a central cancer institute
Anti-cancer propaganda to be divulged across the country	Intense propaganda and education campaign
Creation of regional institutes in other parts of the country linked to local faculties of medicine	Installation of anti-cancer centers in state capitals, preferably annexed to general hospitals
Training for staff and specialization of physicians working for the National Network against Cancer, as well as the lay public.	Education for non-medical workers; education for non-specialized physicians; creation of specialization courses

Source: Andrade, Lana (2010, p.121).

Kroeff undoubtedly emerged as a prominent figure in the quest to establish a centralized policy to combatting cancer, while Prudente personified the approach that placed the state apparatus under the coordination of local organizations. For Kroeff, it was the federal government that should take responsibility for and leadership in this fight, while Prudente felt it should have a secondary role vis-a-vis private initiative.

As it turns out, neither of the plans was executed in full. Throughout the 1940s, the National Cancer Service faced financial difficulties, which left it in a weakened state. Although the anti-cancer leagues expanded their activities and obtained donations from individuals, the support they received from the Union was more political and symbolic than concrete – a situation that was only reversed in the mid-1950s. In contrast, throughout the 1940s, the APCC grew dramatically and managed to raise funds for the construction of A.C. Camargo Hospital, which opened in 1953. This feat received widespread coverage in the São Paulo press, where it was depicted as “the beginning of a new era for the resolution of cancer in Brazil” (Inaugurado..., 25 abr. 1953, p.11). As a result, Prudente gained prominence as one of the leading figures in Brazilian cancerology.

The 1950s was a remarkable decade not only for the APCC and Prudente, but for São Paulo in general. The state was proclaimed by its representatives as proof that nations such as Brazil had the potential to join the ranks of the industrialized world. São Paulo city became the largest city in the country, surpassing the population of Rio de Janeiro, and the state was home to more industrial facilities than anywhere else in Latin America. This feat inspired an extensive calendar of celebrations, shows, and cultural events to celebrate the fourth centenary of the founding of São Paulo city (Weinstein, 2015, p.231).

Against this backdrop, “bandeirante medicine” spoke out about medical specialties in the midst of contentions over the construction of a progressive São Paulo in tune with the new times. One of these specialties was cancerology itself, whose first specialists graduated from their residency, which they undertook at A.C. Camargo Hospital, in 1955. Furthermore, in January 1954, during Getúlio Vargas’s second term (1951-1954), Prudente was appointed director of the National Cancer Service, succeeding its creator and founder, Mário Kroeff, after his dismissal by the Minister of Health, Miguel Couto Filho. Thus, during the fourth centenary of São Paulo came the opportunity to extended the São Paulo project to the whole of Brazil.

His first measure as director was to separate the premises of the National Cancer Service and the Cancer Institute. The former was moved to the 17th floor of the Engineering Club, a building that housed several departments of the new Ministry of Health, while the latter remained at the Gaffrée and Guinle Foundation. The next step was the construction of three new hospitals, located in Uberaba, Curitiba, and Porto Alegre.

As part of his strategy, Prudente declared May to be the month for fundraising campaigns in every state in the country, continuing the pattern adopted since 1946 as leader of the APCC. In July, he organized the sixth International Cancer Congress, held at the Industries Pavilion in Ibirapuera Park. This event attracted cancerologists from all over the world, with representatives from 52 countries and almost a thousand attendees, conferring especial prestige to the proceedings. It was an unprecedented opportunity for Brazilian researchers to engage in the global discussions on the topic.

In short, the decision to appoint Prudente the director of the National Cancer Service was not random, but the outcome of political disputes dating back to the previous decade, culminating in the symbolic year of 1954. However, his tenure at the National Cancer Service was very short-lived. When President Vargas took his life in August of the same year, the Minister of Health and all his appointees were removed from office. Prudente, who had not even completed a year on the board, was replaced by the Rio-based surgeon Ugo Pinheiro Guimarães, who held the position during the administration of President Café Filho.

Although this analysis does not address Guimarães’s work at the National Cancer Service, it is interesting that even though he was aligned with Mário Kroeff, he was still forced to adopt at least some of the measures proposed by Prudente, in harmony with the discussions that took place at the International Cancer Congress. Thus, when writing about the organization and execution of the “anti-cancer fight in Brazil,” the new director stated that:

The isolated action, as it were monopolized [by the State regarding the cancer problem], would be impracticable among us, given the extent of the campaign and the acknowledged material inability of the government to bear the entire inevitable burden.

One or another state government has considered it advantageous to set up its own local bodies [reference to Prudente]. However, the general tendency has been to honor the work of the private organizations that have been founded. This same official state action, when it exists, seeks to act in cooperation with the private philanthropic entity that operates in the State (quoted in Teixeira, Fonseca, 2007, p.87).

This passage suggests a synthesis of the controversies seen in the 1940s, as Guimarães recognized the challenges Brazil faced in its endeavors to fight cancer, due to the sheer size of the country and the government’s financial limitations. This recognition meant local entities and state governments were free to take their own steps in addressing the problem of cancer in their respective states, provided they were aligned with the National Cancer Service, establishing a justifiable partnership between the public and private sectors in addressing the cancer issue.

Thus, the 1950s witnessed the emergence of some important hospitals specializing in cancer, located in the states of Bahia, Rio Grande do Sul, Minas Gerais, Espírito Santo, Alagoas, Piauí, Paraná, Sergipe, and Ceará, as well as the aforementioned A.C. Camargo Hospital, in São Paulo. These institutions were linked to leagues and associations engaged in the fight against cancer, which were in turn registered with the National Cancer Service and the Ministry of Health. Finally, in 1957, the National Cancer Institute (Inca) was opened in Rio de Janeiro, as part of the national development policy implemented by the government of Juscelino Kubitschek. The president called on the country to “take action against ‘chronic, degenerative diseases’ or diseases of the developed world, such as cancer” (Hochman, 2009, p.314-315).

As a result, during the 1950s, historical and political contingencies in Brazil imposed limits on what Prudente could do as director of the National Cancer Service. Despite his aborted tenure, his proposal for a decentralized, networked approach to combating cancer was echoed by his successors, even if this was largely driven by material constraints vis-à-vis the size of the country rather than any ideological affinity or effectiveness of planning. This partial adoption reflects the influence of Prudente’s ideas on the fight against cancer in Brazil, as well as the limitations inherent to the functioning of the National Cancer Service.

## Final considerations

This study revealed that the twenty-year period between 1934 and 1954 saw the creation of an action plan to fight the spread of cancer in São Paulo state, which developed and gained the support of several leading figures and the press, extending beyond its initial geographical remit to become a plan for a nationwide anti-cancer policy. Antônio Prudente was the figurehead of this movement and lent his name to the plan he originally envisaged.

The first part of this article presented the founding of the APCC, which, far from being an isolated initiative, worked in a similar way to other leagues from the same time. What made it different was the format it took: first, both Antônio and Carmen Prudente used representations of cancer to build an effective network of relationships and patronage; second, the APCC was configured as a private fight against a public evil, to the detriment of the government itself, even though it took advantage of São Paulo’s health infrastructure and was justified by the risk that cancer posed; and third, this process was marked by a strong appeal to the sentiment of “Paulistaness.”

The second part of the article focuses on the fundraising strategies the Prudentes employed, as well as the contingencies that favored them in this process. Similarities with the French experience in the interwar period are shown, when the call to fight cancer took on patriotic overtones. In both cases the effect was vehement and passionate mobilization, which was crucial for the success of the initiatives. In São Paulo, this process was materialized in the creation of the A.C. Camargo Hospital, in 1953, celebrated in the São Paulo press as “a new era in the resolution of cancer in Brazil” (Inaugurado..., 25 abr. 1953, p.11).

The final part of the article explores the disputes between cancerologists from São Paulo, led by Prudente, and Rio de Janeiro, led by Kroeff. Notably, previous research on the topic has neglected the ideological substrate of such disputes. We argue that Prudente’s assumptions fit into a liberal ideological spectrum that was characteristic of São Paulo at the time, and were materialized in an action plan known informally as the Prudente Plan. Its main characteristic was the establishment of a decentralized anti-cancer network, which, by reducing Union spending, lent state governments considerable autonomy, not to mention the private leagues and associations, and put placed great faith in voluntary donations by the population.

Although Antônio Prudente took over the leadership of the National Cancer Service in 1954 and had the opportunity to implement the National Cancer Network, his early departure from the position, in the wake of Vargas’s suicide, prevented the complete realization of his ideas. However, his influence continued among his successors, even if it was motivated more by material needs and limitations in meeting national demands than by any ideological alignment with the proposal of a São Paulo plan that sought to be a reference for the whole of Brazil.

## NOTES

1 The documentation used in this study consists of correspondence between the historical agents cited in the text, official state decrees, doctoral theses (which today would be the equivalent of final assignments for degree courses) submitted by students of the Faculty of Medicine of São Paulo, items published in *O Estado de S. Paulo*, and publications (books and articles) from the medical academic world. We retrieved the material from: the Center for Research and Documentation of Contemporary History of Brazil at Fundação Getúlio Vargas (https://www18.fgv.br/cpdoc/acervo/arquivo); the collection of rare works at the Faculty of Medicine, University of São Paulo; the Estadão newspaper archives (https://www.estadao.com.br/acervo/); and the archives of the Library of the Faculty of Public Health, University of São Paulo.

2 The term *bandeirante* was used to refer to people involved in the exploration and exploitation of the land and resources of São Paulo and other regions of Brazil during the sixteenth and seventeenth centuries, often driven by the pursuit of indigenous labor, precious metals, and territorial expansion. Later, in the nineteenth and early twentieth centuries, the São Paulo elite revived the term to forge an ideological narrative that emphasized the pioneering spirit of the *bandeirantes* as a foundation for regional identity and leadership within Brazil.

3 “Paulistaness” is a concept that originated in the historiography of São Paulo, coined by Alfredo Ellis Jr. in his 1934 book, *Populações paulistas*. Encapsulated in the word is a set of values and mindsets associated with the residents of São Paulo, particularly the state’s ruling classes, whose most visible representatives were the “respectable colonels” from inland parts of the state. Ellis Jr. wrote his book at a time of constitutional strife, and he was full of praise for the São Paulo patriots “who volunteered for our constitutionalist army” (Ellis Júnior, 1934, p.153). Subsequently, “Paulistaness” was incorporated into the historiography of São Paulo more critically, with its jingoistic overtones being understood as revealing an ideology that associated the political and cultural virtues of the “São Paulo populations” with the economic and demographic growth seen in the state in the first half of the twentieth century. André Mota (2005), in his doctoral thesis, analyzes how this ideology was manifested in the medical field, exposing the “missteps of *bandeirante* medicine.” Messora (2017), in turn, demonstrates how this ideology was expressed in São Paulo’s desire to take the forefront in cancer treatment, especially between 1920 and 1930, mainly because it was a disease associated with “great civilizations.”

4 In a context in which the main public health concerns were related to rural endemic diseases, it makes sense that cancer, whose etiology was still unclear, should be included under the umbrella of the Inspectorate of Leprosy, Venereal Diseases, and Cancer – a branch of the National Department of Public Health, created in 1919. In São Paulo, there had been a State Sanitation Service since 1892, but it was only aligned with the inspectorate in the 1920s, especially after the Carlos Chagas Reform (1921), so that cancer surveillance fell to the General Disinfection Service and the Demographic and Sanitation Statistics Section (Teixeira, Fonseca, 2007).

5 In 1935, Brazil was divided into federative units, which corresponded to twenty states and the Federal District, which at the time was the city of Rio de Janeiro.

6 In France, as of 1918, women such as the Duchess of Uzès and Baroness Mathilde called on public figures and influential people of their acquaintance to collaborate with voluntary work and generous donations to French health services against cancer (Pinell, 2004). In Rio de Janeiro, women’s leagues emerged from 1953 onwards, but these were more associated with cancer-related educational activities than with fundraising for the construction and maintenance of hospitals. This format was justified for two reasons: first, these initiatives were based on the American Cancer Society, which already brought together countless women to promote health education; second, because techniques to prevent “female cancers” expanded in Brazil during this period (Teixeira, Porto, Noronha, 2012, p.87).
