# Supplementary material for: The “Prudente Plan:” a São Paulo plan for the fight against cancer in Brazil, 1934-1954
Source: Hist Cienc Saude Manguinhos. 2024 Dec 16;31:e2024060. doi: 10.1590/S0104-59702024000100060en (PMC11654734; doi:10.1590/S0104-59702024000100060en)
Supplement: Translation

# Translation: “Plano Prudente”: um projeto paulista de combate ao câncer para o Brasil, 1934-1954

**DOI:** 10.1590/S0104-59702024000100060

**Authors:** Elder Al Kondari Messora, André Mota

**Affiliations:** i Doutorando, Departamento de Medicina Preventiva/Faculdade de Medicina/Universidade de São Paulo. São Paulo – SP – Brasil elderakm@hotmail.com; ii Professor, Departamento de Medicina Preventiva/Faculdade de Medicina/Universidade de São Paulo.São Paulo – SP – Brasil. a.mota@fm.usp.br

**Keywords:** Câncer, Oncologia, São Paulo, Filantropia, História das doenças

## Abstract

Este artigo investiga as estratégias de Antônio Prudente na concepção de uma iniciativa destinada a combater o câncer em todo o território nacional, com base em sua experiência no estado de São Paulo. Para isso, adotamos uma abordagem que combina produção historiográfica com análise de fontes primárias, abrangendo o período entre 1934 e 1954, desde a fundação da Associação Paulista de Combate ao Câncer até a nomeação de Prudente como diretor do Serviço Nacional do Câncer. O estudo destaca as disputas entre os cancerologistas da União e de São Paulo, evidenciando a singularidade da proposta paulista na política anticâncer, marcada pela associação a ideais liberais e pelo sentimento de “paulistanidade” que a permeia.

Enaltecido por seus dirigentes, no alvorecer do século XX, o estado de São Paulo era propagandeado como uma “sociedade livre”, de “economia competitiva”, cujo “ouro verde” havia emergido tal como dínamo do progresso e da independência. Uma referência à prosperidade da lavoura cafeeira, uma vez que, desde 1840, o café tinha se tornado o principal produto de exportação do Brasil. Sua expansão deu origem a um grupo com poder econômico suficiente para disputar o domínio político do país, inclusive com os representantes do poder central. Além disso, os crescimentos urbano e demográfico, que se intensificaram com a imigração, impulsionaram problemas de saúde, conforme indicados nos registros sanitários.

Uma dessas patologias foi o câncer, doença misteriosa que nem sequer aparecia nos anuários demográficos paulistas no começo do século XX, tratada como um pequeno achaque entre tantos outros e sem qualquer relevância. No entanto, com o passar do tempo, o número de óbitos associados aos tumores malignos aumentou de maneira significativa, tornando-se uma questão de saúde pública que não poderia ser ignorada.

A partir de 1920, surgiram as primeiras organizações paulistas dedicadas a lidar com o problema, com destaque para a fundação do Instituto Arnaldo Vieira de Carvalho e, anos mais tarde, da Associação Paulista de Combate ao Câncer (APCC), criada em 1934. Sob um contexto mais abrangente, médicos da Bahia estudavam o câncer desde 1840, embora seus interesses sobre o assunto estivessem associados ao diálogo com as produções científicas francesas, e não com a quantidade de pessoas afetadas na região. A Liga Bahiana Contra o Câncer foi fundada em 1936, fato que evidenciou a falta de atenção dos poderes públicos e da sociedade a essa questão durante um longo período (Souza, 2014).

A Reforma Carlos Chagas, de 1921, desempenhou papel fundamental quanto ao aprimoramento da visibilidade do câncer. A instauração do Departamento Nacional de Saúde Pública tornou obrigatória a notificação dos casos à Inspetoria de Profilaxia da Lepra e das Doenças Venéreas, contribuindo, dessa forma, para o mapeamento progressivo dos diagnósticos. Nesse período, merecem menções as inaugurações do Instituto de Radiologia do Rio de Janeiro, em 1919, e do Instituto do Radium de Belo Horizonte, em 7 de setembro de 1922 (Teixeira, 2010). Ademais, decorridos alguns anos, foi criada a Liga Brasileira Contra o Câncer, e, no ano de 1935, realizou-se o primeiro Congresso Brasileiro de Câncer.

O histórico dessas iniciativas evidencia como a preocupação com a doença cresceu em âmbito nacional, demonstrando a coerência e a convergência de alguns compromissos em diferentes estados. Cumpre-nos, agora, compreender suas particularidades relacionadas a São Paulo, e, para isso, este artigo foi dividido em três partes.

Na primeira, apresentamos a fundação da APCC, engendrada pelo cirurgião Antônio Prudente, que aproveitou a estrutura sanitária instituída em São Paulo para enfrentar a iminente questão de saúde pública que o câncer representava. Na segunda, abordamos as estratégias de Prudente e de sua esposa na construção de uma rede filantrópica e facultativa de luta contra o câncer, comparando-as às experiências do Rio de Janeiro e da França. O corolário da missão paulista culminou na construção do Hospital A.C. Camargo, no ano de 1953. Por fim, na terceira parte, explicamos as bases intelectuais e ideológicas do “Plano Prudente”, um “plano nacional paulista” de combate ao câncer, que causou tensões políticas entre renomados cancerologistas do Distrito Federal. Esta última se encerra com a designação de Antônio Prudente ao cargo diretivo do Serviço Nacional do Câncer (SNC), em 1954, que, apesar de uma experiência curta, deixou marcas e influências.

Para esclarecer a abordagem deste trabalho, nossa pesquisa está inserida nos estudos dedicados à história do combate ao câncer no Brasil, com ênfase especial no estado de São Paulo, durante um período de duas décadas, de 1934 a 1954. Nossa análise abrange informações que ultrapassam esse intervalo, uma vez que acreditamos que eventos históricos estão interligados por uma “dialética das durações” (Braudel, 1965, p.289), e que a compreensão de certos fatos depende da interação com outros eventos de médio ou longo prazos.

A condução da pesquisa envolveu levantamento de fontes primárias^1^ que foram contextualizadas no âmbito da historiografia do câncer, em que destacamos as contribuições de Bodstein (1987), Bertolli Filho (2002), Teixeira e Fonseca (2007). Para análise do conteúdo, utilizamos os conceitos de representação (Chartier, 2002), filantropia (Sanglard, 2010), medicina bandeirante (Mota, 2005) e as noções de rede local permanente e vertical permanente especializada (Merhy, 1992).

Tal abordagem metodológica e teórica permitiu-nos compreender como a proposta paulista de combate ao câncer, promovida pelo casal Prudente, é atravessada por contingências históricas mais amplas, tanto de âmbito nacional quanto internacional, estando intrinsecamente ligada às ideias liberais e impregnada por um forte sentimento de “paulistanidade”^2^ (Ellis Júnior, 1934; Mota, 2005; Messora, Mota, 2018).

## Presságio de guerra: Antônio Prudente e a origem da Associação Paulista de Combate ao Câncer

Na primavera de 1933, o jornal *O Estado de S. Paulo* publicou uma série de artigos sobre câncer, com autoria de Antônio Prudente Meirelles de Moraes (1906-1965), um médico que se formou na Faculdade de Medicina de São Paulo, em 1928. Pouco tempo após obter seu diploma, Prudente embarcou para a Alemanha, com o objetivo de se tornar aprendiz do professor Franz Keysser, um médico alemão que havia conquistado notoriedade internacional após elaborar seu aparelho de bisturis elétricos para lidar com tumores até então inoperáveis.

Muitos médicos brasileiros baseavam-se em seus postulados, inclusive Prudente, que, ao voltar ao Brasil, se tornou assistente de técnica cirúrgica da Faculdade de Medicina e, em 1935, ingressou na Escola Paulista de Medicina como professor catedrático de cirurgia reparadora e plástica. Não obstante, o que fez com que o neto do primeiro presidente civil do país garantisse um lugar privilegiado nesta narrativa foi outra situação: seus cinco artigos no jornal *O Estado de S. Paulo*, posteriormente reunidos no livro *O câncer precisa ser combatido* (Prudente, 1935). Essas publicações pressagiaram suas iniciativas contra a doença no estado de São Paulo e apresentaram Prudente como uma autoridade no assunto.

Seu primeiro texto discorreu sobre o recrudescimento mundial das pesquisas relacionadas a tumores malignos, o que contrastava com a situação no Brasil, que ainda engatinhava nesse campo. De acordo com Prudente, essa disparidade ocorria devido à aparente insolubilidade dos mistérios do câncer, fundada no “desconhecimento quase completo por parte do nosso povo” (O problema..., 13 out. 1933, p.6). No entanto, enfatizava que a cura era possível, se o diagnóstico fosse precoce. Assim, fazia-se necessária uma campanha que se tornasse capaz de fazer desaparecer “preconceitos estúpidos e muitas vezes prejudiciais” (p.6).

No que diz respeito a esse caso, Prudente referia-se à “cancerofobia”, disseminada por teorias que buscavam encontrar a “regionalidade cancerosa” (Portugal, nov. 1910a, p.35) ou identificar as “ruas ou casas do câncer” (Ramos, set. 1911, p.163-164). Essas teorias se baseavam em observações de movimentos concêntricos, partindo de indícios obituários e afunilando-se até a menor escala considerada, conhecidas como “casas malditas” (Portugal, nov. 1910b, p.44; Ramos, set. 1911, p.163-164). A percepção de que esses lugares seriam fontes de propagação do câncer não poderia, de forma alguma, ser subestimada, de modo que chegou a ser uma prerrogativa da Inspetoria da Lepra, Doenças Venéreas e Câncer.^3^

Desde o século XIX, jornais paulistas divulgavam pesquisas que reforçavam essa percepção, com destaque para aquelas realizadas por Domingos José Freire Junior, afirmando ter descoberto o “micróbio do câncer” (Messora, 2017, p.62). Embora o médico tenha caído em relativa obscuridade, suas ações pioneiras contra o câncer estão inseridas em uma perspectiva compartilhada por inúmeros pesquisadores da época, que era a necessidade de investigar experimentalmente a transmissibilidade das doenças. Pedro Severiano de Magalhães foi um dos que partilharam tais concepções, publicando, em 1888, a *Teoria parasitária do câncer*. Freire, por sua vez, desenvolveu uma vacina contra os tumores malignos, mas que não chegou a ser aplicada em muitas pessoas.

Na ausência de uma definição etiológica, os cientistas de São Paulo sugeriam cautela diante do risco de contágio. O médico Olympio Portugal (nov. 1910a, nov. 1910b), membro da Sociedade de Medicina e Cirurgia de São Paulo, foi uma das vozes que reforçaram essa percepção, comparando a doença à tuberculose. Nesse contexto, não demorou para que as “casas malditas” fossem alvos de medidas profiláticas do Serviço Geral de Desinfecção, mesmo sem comprovações científicas definitivas. Tratava-se, antes, de um “gerenciamento de dúvida” por parte das autoridades públicas (Araújo Neto, 2019).

O impacto dessa representação estendeu-se para além da década de 1950, associada à teoria miasmática, e, assim, tornou-se comum pessoas atravessarem a rua antes de estarem próximas ao hospital A.C. Camargo. Para nós, é sintomático que, durante sua construção, a instituição tenha enfrentado denúncias por se localizar em uma região populosa da cidade. Nesses momentos, Prudente ia aos jornais reafirmar: “Um dos absurdos que se propala é ser o câncer uma moléstia contagiosa, não devendo ficar os cancerosos num hospital tão central. O cancer não é contagioso, de acordo com as conclusões de todos os Congressos Internacionais, realizados até hoje. Se alguém assim o considera, deveria, a meu ver, demonstrá-lo imediatamente para bem de toda a humanidade” (Notícias..., 27 jun. 1944, p.5).

Prudente demonstrava certo apreço pela teoria embrionária e, em seu segundo artigo, argumentou que os tumores se originavam de malformações ou mutações nos tecidos durante sua fase de desenvolvimento, por isso exigia de seus pacientes atenção às cicatrizes, verrugas, cáries e papilomas. Essa perspectiva ganhou força à medida que outras doenças, como a febre amarela, tuberculose ou tracoma, reduziram seus índices após intervenções dos sanitaristas. O câncer, por outro lado, crescia vertiginosamente.

O terceiro artigo foi dedicado às estatísticas sobre a doença, quantidade de óbitos e distribuições etnográficas. Essa última se referia ao “grau de civilização dos diferentes povos” (O problema..., 9 nov. 1933, p.4). Entre médicos, Prudente não era o único a afirmar que “o câncer é mais comum nos países civilizados do que [ilegível] dos povos primitivos” (p.4), além de ratificar que a doença acometia muito mais mulheres que homens. Ao final do texto, o cirurgião apresentou aos leitores a categoria de “câncer profissional”, que nada mais é do que a doença relacionada às profissões que os operários exerciam.

No quarto e no quinto artigos, o médico paulista explicou como a doença evoluía, as causas de morte, os sintomas, o que era necessário para preveni-la, entre outras situações que, na verdade, confluíram para um único caminho: a defesa de uma organização anticancerosa em São Paulo (Prudente, 1935).

A valer, as contingências históricas favoreceriam a empreitada que Prudente almejava. Desde a década de 1890, quando, sob a nova ordem republicana, as questões relativas à saúde pública passaram para a alçada dos estados, São Paulo reformulou sua estrutura organizacional. No campo da saúde, essa mutação se materializou com a construção do primeiro Código Sanitário, em 1894, e a organização do Serviço Sanitário, entre 1891 e 1892. O segundo subordinava-se à Secretaria do Interior e contava com uma série de órgãos, como a Diretoria do Serviço Sanitário, o Hospital de Isolamento, o Hospício de Alienados, o Serviço Geral de Desinfecção, os Laboratórios de Análises Químicas e Bromatológicas, Químico e Farmacêutico; os Institutos Vacinogênico e Bacteriológico, além da Seção de Estatística Demográfico-Sanitária. Pouco depois foi criado o Instituto Butantã e inaugurado o Juquery, voltado para as doenças mentais (Mota, 2005; Escorel, Teixeira, 2008).

A literatura informa que todo esse aparato não encontrava equivalência em outro estado da federação, e seu custo consumia porções significativas do orçamento paulista (Ferreira, Luca, 2011), porém, esse foi o modo que seus governantes propuseram para lidar com os problemas de saúde, sobretudo os elevados índices de malária e febre amarela que assolaram Santos e Campinas, além da varíola na capital. Não suficiente, ainda foram registrados casos de peste bubônica, cólera e infecções gastrointestinais, questões que, de algum modo, estavam associadas à expansão urbana e ao crescente desenvolvimento socioeconômico que vivia a região (Ribeiro, 1993).

O caráter contagioso dessas doenças impôs a necessidade de uma autoridade capaz de atuar sobre todo o território e sua população, mesmo que tais medidas pudessem restringir certas liberdades individuais ou violar autonomias políticas e até direitos de propriedade, principalmente quando os doentes, em sua maioria classificados como pobres, representavam uma ameaça coletiva devido ao potencial transmissivo de seus males para as classes abastadas (Hochman, 1998).

O aparato paulista que se constituiu foi uma resposta a isso e a seus entraves, e é sob essa conjuntura que o câncer emergiu como uma questão em pauta, o que também atribui sentido às iniciativas de Prudente. À medida que a capital paulista se posicionava como baluarte de ações modernizadoras, de acordo com as conquistas científicas do período, não causa estranhamento a “medicina bandeirante” (Mota, 2005) colocar-se à disposição para enfrentar uma moléstia que recebia a alcunha de “doença das grandes civilizações”.

Na década de 1920, foi construído o primeiro hospital voltado aos pacientes com câncer, uma iniciativa de Arnaldo Vieira de Carvalho. Em seguida, Antônio Prudente assumiu o protagonismo dessa luta, a qual se mostra como um momento-chave de consolidação do substrato ideológico de sua causa, porque, em relação às ações estatais quase nulas na década de 1930 (Teixeira, Fonseca, 2007), o “problema do câncer em nossa terra só pode ser resolvido dentro das condições de vida de cada unidade federativa e de acordo com as possibilidades materiais e intelectuais dessas mesmas unidades”, informava Prudente (1935, p.168).

Sua proposta era a de que os órgãos públicos agissem urgentemente sobre a questão e que fosse criada a Inspetoria de Combate ao Câncer, nos mesmos moldes da já existente Inspetoria de Profilaxia da Lepra. Entretanto, como a ênfase das políticas públicas recaía sobre a luta contra as endemias rurais e as epidemias que ameaçavam a população, “infelizmente” – lamentava o médico – “não se cogita da criação desse novo departamento do Serviço Sanitário” (Prudente, 1935, p.172). Restava, portanto, que a luta contra os tumores malignos fosse dirigida pela iniciativa privada, e, em vista disso, o cirurgião assumiu para si essa missão. Assim, em 1934, foi fundada a entidade filantrópica APCC, que, em sua opinião, “representava as necessidades do Estado no problema do câncer” (p.176).

Como a primordialidade de um diagnóstico precoce para o tratamento fazia parte do consenso médico, tal organização ficou responsável por sanar os dois problemas que obstaculizaram a profilaxia no estado: “A completa ignorância, por parte dos leigos, dos dados relativos à moléstia, e a deficiência de conhecimentos com respeito ao câncer, por parte de médicos, dentistas, farmacêuticos, parteiras, enfermeiros etc.” (Prudente, 1935, p.171). Para atender a essa demanda, foi necessária uma propaganda “anticancerosa” eficaz, que foi lançada por meio de cartazes, panfletos, jornais, cinematógrafos e rádios, a fim de ampliar o conhecimento e, assim, facilitar a descoberta precoce da moléstia.

Essas diretrizes, não obstante, estavam em consonância com as de outras ligas surgidas no mesmo período, mas o que marcou a iniciativa de Prudente pode ser identificado a partir de três situações: a primeira diz respeito às estratégias e retóricas adotadas pelo médico paulista e sua esposa, Carmen Prudente, pois souberam tirar proveito das representações do câncer e de uma ampla rede de relacionamentos importantes; a segunda resume este tópico: em meados da década de 1930, consolidou-se uma associação de caráter filantrópico, aglutinando as diligências e os cuidados no que diz respeito ao câncer, formalizando a luta dos paulistas contra a doença, por um viés que compreende a “política de saúde pública” de forma privada. Por fim, a terceira refere-se à circunstância propícia sobre a qual o casal efetivou suas estratégias, mobilizando o sentimento da população tal como a experiência francesa em que se basearam.

## Filantropia e paulistanidade: as estratégias do casal Prudente e a fundação do Hospital A.C. Camargo

Em fins do século XIX, cresceu no meio médico a percepção de que uma série de problemas, principalmente aqueles decorrentes da pobreza, não poderia ficar à espera de uma pequena parcela caridosa da população. Influenciados pelos franceses, os médicos brasileiros passaram a defender a necessidade de se organizar no país formas de assistência pública, planejadas e postas em prática pelo governo por meio de estrutura institucional, pessoal técnico e medidas racionalizadas, de acordo com os fundamentos científicos necessários (Martins, 2011).

Assim o fez Antônio Prudente, que, sob a mesma influência, afirmava:

Nós poderíamos fazer coisa semelhante, principalmente no Estado de S. Paulo, onde o meio já comporta tal organização. Aliás, o nosso Serviço Sanitário está tão bem aparelhado que não seria difícil conseguir para todo o Estado, aproveitando mesmo material e funcionários, uma organização perfeita, não só no ponto de vista de pesquisa e tratamento, como também no que se refere à propaganda anticancerosa.

... Estou certo que com o auxílio da própria população e fazendo parte do Serviço Sanitário do Estado em pouco tempo estaríamos na altura dos países mais avançados (O problema..., 7 jan. 1934, p.6).

O poder público, contudo, não comprou sua ideia, e isso o levou a fundar a APCC, em 1934. Mas a ambição da associação ganhou contornos bem delineados apenas na década seguinte, quando recebeu uma doação de terreno do governo do estado de São Paulo. Para formalizar o donativo, estiveram presentes o interventor federal Fernando Costa e o diretor do Instituto do Câncer de Buenos Aires, Angel Roffo, que, além de renomado cientista, havia criado, junto com sua esposa, Helena Larroque, uma associação civil que organizou a luta contra o câncer na Argentina, em 1921 (Buschini, 2014).

Planejava-se, doravante, construir o Instituto Central do Câncer – posteriormente denominado Hospital Antônio Candido de Camargo –, uma organização que representou o elo entre as concepções morais da caridade cristã e a filantropia, isto é, um modelo racionalista e público que ganhou terreno nas primeiras décadas do século XX em todo o país (Sanglard, 2010). Agregavam-se ainda os avanços da medicina, as concepções de câncer, as organizações de saúde pública e as possibilidades materiais para sua expansão. Um emaranhado que atribui sentido para a atuação de homens e mulheres de classes abastadas na incursão de práticas assistenciais que, no caso da luta contra o câncer em São Paulo, passavam pelas mãos de Antônio e Carmen Prudente.

Se o primeiro foi hábil em aglutinar o interesse de vultosos filantropos para sua causa, como no caso do comendador Martinelli, que realizou uma doação de cinco milhões de cruzeiros à APCC (Martinelli, 29 dez. 1943), sua esposa foi além. Em 1946, Carmen fundou a Rede Feminina de Combate ao Câncer, uma organização que promoveu eventos festivos de diversas ordens e “manteve um cadastro contendo o número telefônico de grande parte da elite paulistana, cujos membros eram mensalmente convidados a contribuir para a campanha em prol de um hospital na cidade. A adesão à entidade era tal que ela conseguiu reunir em suas hostes mais de 25 mil mulheres” (Teixeira, Porto, Noronha, 2012, p.51) e deu início a um movimento de ligas femininas que se espalhou por todo o país, principalmente a partir da década de 1950.

Nesse período, as mulheres estavam majoritariamente excluídas do mundo público, porque a sensibilidade burguesa que se constituiu no começo do século XX se deu em torno de uma forte separação ideológica entre as esferas domésticas e dos sentimentos e a esfera pública e da racionalidade. Entretanto, foi a partir da exclusão das mulheres do mundo público que a filantropia terminou associada à atividade feminina, tendo em vista que – pensavam seus contemporâneos – mulheres estariam mais aptas a fazer o bem e a prestar cuidados aos necessitados, além de terem tempo livre para organizar associações benemerentes e angariar recursos para as boas obras (Martins, 2015). É sob essa ótica que a prática filantrópica de Carmen Prudente emergiu como um dispositivo de intersecção entre as esferas.

Foi durante o Estado Novo que as senhoras endinheiradas se sentiram habilitadas a intervir socialmente, e o trabalho exercido por Carmen e por outras mulheres não atentava contra a moral ou a virtude, pelo contrário, propiciava condições para o exercício de uma “cidadania feminina”, baseada na utilidade social das mulheres: a vocação maternal (Martins, 2011). Curiosamente, o ideário de que a capacidade de cuidar seria uma extensão natural da própria maternidade valeria mesmo para aquelas que não eram mães: “não tivemos filhos”, costumava dizer Carmen, “exceto um, de concreto”, referindo-se ao hospital (Bueno, 2015).

Essa ação do casal Prudente foi bem-sucedida e aproveitou uma oportunidade de caráter substancialmente simbólico para afirmar seus objetivos: o sentimento de “paulistanidade” – ideia que pode ser traduzida na narrativa construída e bem disseminada de que “os bandeirantes não viam peias para as suas empresas temerárias” e venceriam “todos os obstáculos” (Ellis Júnior, 1926, p.353). Não por acaso, a estratégia retórica do casal abusava dos elogios ao povo de São Paulo, de patriótica iniciativa e colaboração abnegada, já que “os governos estadual e federal pouco contribuíram para tão grande obra, pois não chegaram sequer a dar 10% do total das doações da população paulista, as quais ultrapassaram a casa dos 30 milhões de cruzeiros” (Iniciada..., 22 maio 1952, p.11).

Para alcançar esses números, uma série de eventos foi promovida, como a “Campanha do tijolo e do cimento” e o “Dia de trabalho”. Este, por exemplo, propunha que os funcionários das fábricas doassem o valor correspondente a um dia de expediente. Além dessas, foram realizadas exposições “anticancerosas”, com ingressos pagos, além de bailes, bilhetes de cinema e até jogos de futebol. Entre as senhoras da Rede Feminina acontecia a chamada “Corrida do caranguejo”, que consistia em premiar com um caranguejo de ouro quem arrecadasse a maior quantia em donativos, incentivo realizado por uma joalheria famosa da época (Nenhum..., 18 maio 1948, p.2).

Dentro da própria APCC foi fundado o grupo “Comandos contra o câncer”, voltado para investidores privilegiados, tais como industriais, comerciários e pessoas de posses. Seus participantes recebiam patentes do exército de acordo com a quantia de suas doações, atualizadas a cada encontro dos comandos, ocorridos mensalmente (Campanha..., 19 jun. 1952, p.11). Em paralelo ao evento “militar”, acontecia a expedição “Toalha mágica”, que consistia na divisão de um longo tecido em 32 partes distribuído entre as esposas dos “comandantes” e “oficiais”. Em posse da toalha, as senhoras precisavam buscar assinaturas para seus pedaços, e cada rubrica valia mil cruzeiros, podendo cada pedaço conter trinta nomes. Essa estratégia também colheu bons frutos, e novas toalhas, a preços acessíveis, começaram a ser produzidas pela Rede Feminina (Campanha..., 28 maio 1952, p.11).

Vemos, portanto, práticas que foram arquitetadas para combater uma doença sob a concepção de um problema de saúde pública, porém, com papel modesto por parte dos poderes públicos. O apelo ao patriotismo paulista era uma das estratégias que nutria certa determinação popular nessa luta. Merecem menção também as forças da representação ou metáforas do câncer (Chartier, 2002; Sontag, 1984), o medo que a doença causava e a própria credibilidade que as atividades filantrópicas adquiriram.

Diferentemente da caridade, a filantropia não carregava em si a virtude da piedade, tratava-se, antes, de uma atividade laica e de utilidade social. Para ser posta em prática, era necessária a reunião de pessoas que “participam de um mesmo movimento de expressão e de identidade do doador: elas se remetem às convicções, o situam em um espaço social, o inscrevem no seio de um grupo de relações” (Sanglard, 2010, p.128). Foi o que fez o casal Prudente, mobilizando grupos de apoio para sua causa e agindo diante da lacuna de atuação do poder público. Experiência distinta do que ocorreu no Distrito Federal, mas semelhante ao caso francês, no qual as mulheres tomaram a dianteira do movimento com o intuito de fomentar a construção de hospitais.^4^

O viés solidário de suas ações, entretanto, não excluía nem escondia outras motivações, tal qual o anseio para firmar posições de relevância no cenário acadêmico e profissional (em meio à constituição de uma especialidade como a cancerologia) e a ambição de se inserirem como protagonistas de uma causa com amplitude nacional e internacional.

Com efeito, relações entre instituições de câncer e ações filantrópicas não se iniciaram em São Paulo e eram discutidas de maneira intensa no Rio de Janeiro, onde parte considerável dos filantropos era remanescente da elite oriunda do Império, formada por barões, condes, viscondes, baronesas e viscondessas; porém, foi um notório industrial quem esteve à frente da construção de um hospital do câncer no Distrito Federal, o carioca Guilherme Guinle. Na França não era muito diferente, com a nobreza do *Ancien Régime*, políticos e industriais contribuindo financeiramente para o enfrentamento do flagelo.

No Rio de Janeiro, a atitude de Guinle não ganhou repercussão na imprensa ou mesmo em periódicos especializados. Desse modo, as primeiras plantas do hospital do câncer, desenhadas e apresentadas em 1925, nunca saíram do papel. Dez anos mais tarde, com donativos de poucos particulares e ainda sem qualquer apoio do governo federal, o próprio Guinle desistiria de levar adiante tal objetivo (Sanglard, 2010). Foi apenas com a ascensão de Vargas, isto é, com a centralização das ações no Estado, que o Instituto de Câncer se ergueria.

Do outro lado do Atlântico, na França, a origem da luta contra o câncer remete ao contexto da Primeira Guerra Mundial. O entusiasmo patriótico que floresceu em meio às hostilidades bélicas favoreceu doações caridosas em prol do sistema de saúde francês, à medida que as necessidades de guerra requeriam o esforço de todos os homens no Exército, fossem jovens ou velhos. No entanto, a circunstância etária destes últimos exporia que a incidência de câncer entre os soldados não era tão rara assim, fato que se tornou um problema para as autoridades médicas militares na França (Pinell, 2004).

Essa conjuntura serviu para que os franceses tivessem consciência das dimensões sociais que o câncer retumbava. Logo, quando lideranças sanitárias decidiram agir sobre a questão, a estratégia de trazer para a causa personalidades conhecidas foi posta em prática, mobilizando desde a antiga nobreza até o cidadão comum, todos em solidariedade aos soldados. Em 1918, a Liga Anticâncer Franco-Anglo-Americana legitimou-se, presidida pelo político francês Justin Godart, sob o consentimento dos embaixadores britânico e estadunidense (Pinell, 2004).

Não seria forçoso o paralelo entre a convocação patriótica feita pelos franceses e o chamamento das “populações paulistas” contra o câncer, tendo em vista que, em ambos os casos, a invocação de sentimentos contribuiu significativamente para aumentar a boa vontade cidadã. Assim, o Instituto Central do Câncer (Hospital A.C. Camargo) foi fundado em 1953, fruto de uma aliança promovida entre os médicos, as organizações filantrópicas e o povo paulista. Orgulhoso, Prudente via o Instituto constituir “um anseio do nosso povo, uma aspiração de muitos que contribuíram para sua realização” (Inaugurado..., 25 abr. 1953, p.11). Por outro lado, no Rio de Janeiro, a mobilização não obteve a mesma repercussão nem ampla adesão popular. Diante desse cenário, tornou-se necessário que o governo interviesse de maneira significativa na construção do aparato de luta contra a doença.

Fosse isso suficiente, a história estaria encerrada, mas Prudente manifestava outras ambições: queria que seu projeto se estendesse para todo o país. Assim, durante a ausência do diretor do SNC, Prudente entregou sua proposta ao governo federal, desencadeando querelas com oncologistas que atuavam no Distrito Federal.

## “Plano Prudente”: um projeto paulista de combate ao câncer para o Brasil

A década de 1940 foi marcada por diretrizes centralizadoras do regime de Getúlio Vargas. A saúde pública sofreu algumas alterações, foram instaurados 13 serviços nacionais relacionados à infraestrutura e ao controle de doenças específicas, que eram consideradas prioritárias na época. Entre eles, estava o SNC, criado por meio da lei n.3643, de 23 de setembro de 1941, e submetido ao Departamento Nacional de Saúde, órgão integrante do Ministério da Educação e Saúde.

Importa-nos o fato de que o SNC incorporou definitivamente a doença na pauta das ações de saúde pública para todo o país (Brasil, 23 set. 1941), e seu primeiro diretor foi Mário Kroeff. Com efeito, após sensibilizar Getúlio Vargas para o problema do câncer, o médico gaúcho conseguiu que o presidente assinasse o decreto-lei n.378, de 13 de janeiro de 1937, criando o Centro de Cancerologia – embrião do SNC –, sendo designado, pouco tempo depois, para a direção do serviço. Kroeff não teve como iniciar as atividades que considerava centrais nesse momento, mas se incumbiu de aprender sobre as melhores práticas e se apropriar do que havia de mais avançado em termos de aparatos terapêuticos, viajando aos EUA em 1942.

A APCC, paralelamente, auferia importância suficiente para empreender certa rivalidade com o núcleo de cancerologistas do Distrito Federal. Prudente encaminhava cartas para Getúlio, informando que a União não precisaria sequer ter tantos gastos públicos, exceto na manutenção das unidades de tratamento. Como prova, dizia-lhe que pediria a um renomado patrício de São Paulo doações diretas, e que o presidente poderia até mesmo se aproveitar disso como propaganda política (Prudente, 31 jan. 1943), solicitação que resultou na doação de Martinelli, citada anteriormente.

Sem coincidência, o contato do cirurgião paulista com a alta cúpula do governo federal deu-se justamente quando Kroeff estava em intercâmbio. Ao Ministro de Educação e Saúde, Prudente enviou um projeto intitulado Rede Nacional Contra o Câncer, informando que “a necessidade dessa Rede se impõe diante do abandono quase total em que se encontram os portadores de câncer” (Prudente, 31 jan. 1943).

A característica marcante do projeto estava em sua organização descentralizada, ideologicamente liberal. Prudente dividia o país em várias regiões que deveriam receber centros de tratamento. Opunha-se ao modelo do SNC, pois considerava “uma utopia organizar uma luta contra o câncer excessivamente centralizada” devido à distribuição demográfica desigual no território nacional. Para ele, o combate contra a doença deveria se dar sob o formato de uma rede que abrangesse todas as regiões, estabelecida por unidades distintas (Prudente, s.d., p.2).

Cabe retroceder um pouco para, em nossa análise, compreender as contingências históricas que nutriram e sustentaram a proposta de Prudente.

Desde sua origem, em 1892 (com a fundação do Serviço Sanitário), a infraestrutura sanitária de São Paulo foi organizada e prestada pelos governos municipais e estaduais, com patrocínio de cafeicultores, de modo que a União exerceu pouca influência direta em seus dispositivos.

A partir de meados da década de 1920, as políticas de saúde que se efetivaram foram inspiradas no modelo americano dos Health Centers, difundidos pela Fundação Rockefeller e pela Escola de Higiene e Saúde Pública da Universidade Johns Hopkins, na qual estudou o então diretor do Serviço Sanitário, Geraldo Horácio de Paula Souza (Campos, 2002). Sob sua gestão, que durou de 1922 a 1927, os Centros de Saúde foram espalhados por toda a cidade de São Paulo, e educadoras sanitárias incumbiram-se de ensinar os preceitos básicos de higiene para a população. Esse modelo, intitulado “rede local permanente”, portava um ideário próximo dos projetos liberal-democráticos que, a partir da década de 1930, durante o governo Vargas, entraram em descrédito. A substituição se deu por meio de outro modelo de serviços, chamados “verticais permanentes especializados” (Merhy, 1992, p.27).

O primeiro, “rede local permanente”, supunha uma relação entre a ação estatal e os setores sociais, por meio dos serviços de saúde e assentada sobre uma organização capaz de tratar dos problemas coletivos de saúde, que poderiam ser reduzidos ou sanados por meio da consciência sanitária de cada sujeito. Já o segundo modelo manifestava-se a partir de órgãos públicos que agiam sobre problemas específicos de saúde pública, controlando-os em escala individual e coletiva a partir de atendimentos especializados.

Prudente não estava longe da segunda categoria, se considerarmos que o campo oncológico requeria tecnologias de alto investimento e a especialização tornar-se-ia uma obrigatoriedade. Não é por acaso que, se na década de 1930 os serviços “verticais permanentes especializados” (Merhy, 1992, p.27) foram implantados, também foi nesse período que Prudente passou a escrever nos jornais e fundou a APCC. Entretanto, a orientação para saúde pública de São Paulo desse período ainda se baseava no Código do Serviço Sanitário, reformado de acordo com os Health Centers, sofrendo pouca influência da União, mesmo sob o governo varguista, o que explica a alta rotatividade de diretores que não se firmavam no Serviço Sanitário (Mascarenhas, 1949).

Diante desses condicionantes, compreendemos que a materialização da proposta em rede feita por Prudente nutria-se de ambos os modelos: da aceitação de que é necessária uma especialização sofisticada em cancerologia, portanto, da verticalização dos atendimentos; e adaptada em favor de uma rede distribuída de postos hospitalares e de educação sanitária, em concordância com a “rede local permanente” (Merhy, 1992, p.27).

O caráter liberal materializou-se no estímulo a uma rede autônoma distribuída em unidades, opondo-se a uma organização forte e centralizada. Essa concepção se estendia na forma de financiamento, tendo em vista que o governo teria menos despesas, e o contribuinte individual, um papel facultativo. Foi esse, afinal, um dos argumentos de Prudente para convencer Getúlio quanto à vantagem de seu plano: o médico alvitrou que os fundos necessários para a realização de seu projeto poderiam ser obtidos por meio do apelo à generosidade do povo, propondo uma “Campanha nacional contra o câncer”, sob a epígrafe expressa pelo próprio presidente da República: “É preciso, antes de mais nada, alarmar o povo!” (Prudente, s.d., p.13).

Seu passo seguinte foi o de preconizar uma organização exatamente igual à APCC e à Rede Feminina de Combate ao Câncer, instituída por Carmen, mas para todo o Brasil. Retirava-se, dessa forma, a exclusividade da organização central do SNC, defendendo que:

Em cada cidade que constitui a sede de uma Unidade da Rede, deverão ser organizadas duas comissões. A primeira será formada por médicos e elementos representativos da localidade, sob a Comissão Executiva local da Campanha. A segunda será integrada apenas por senhoras, tendo por fim o trabalho de solicitação e arrecadação de donativos (Prudente, s.d., p.13).

Por fim, Prudente ofereceu-se para assumir a posição de “diretor-técnico” da campanha, com uma atuação que abrangeria todo o território nacional. Essa proposta era particularmente sedutora para Getúlio, uma vez que o Ministério da Educação e Saúde estava concentrado em doenças prevalentes, como a febre amarela e a malária (Teixeira, Fonseca, 2007, p.78). Portanto, a ideia de instituições privadas lidando com a questão do câncer, com modestas subvenções federais e estaduais, parecia mais atraente aos olhos do presidente. Sem demora, ele deu início à campanha nacional.

Já havia, contudo, outra “campanha contra o câncer” em curso, anunciada justamente com a criação do SNC, em tudo semelhante, exceto no peso atribuído ao aspecto voluntariado e contributivo da população. De acordo com o documento fundante da instituição, ao SNC competia organizar, orientar e controlar, em todo o país, uma campanha investigativa, epidemiológica, profilática e terapêutica da doença, bem como de vigilância dos doentes após tratamento ou asilamento dos mais necessitados (Bodstein, 1987), todavia era ausente a ideia de angariar fundos a partir da comoção social causada pela doença. Isso não significava que a instituição não recebesse patrocínio de particulares ou que não contasse com o trabalho de grupos voluntários, mas essas atividades não estavam formalmente prescritas, tampouco eram um objetivo direto ou ocorriam de forma sistemática.

Esse tipo de contribuição ganhou algum destaque no órgão governamental principalmente a partir de 1948, porque Kroeff objetivou “causar impacto na opinião pública, que, a seu ver, estava alheia ao problema do câncer” (Teixeira, Fonseca, 2007, p.98). Para isso, estabeleceu uma parceria com o Jockey Club com a finalidade de elaborar a primeira campanha educativa contra o câncer do SNC. Três anos depois, outro evento favoreceu o aporte de novos recursos e de seu fortalecimento institucional: o médico e vereador paraibano Napoleão Laureano, ao sofrer com câncer terminal, coordenou uma campanha para criação de um hospital do câncer em sua região de origem, proporcionando significativo número de doações para a fundação do Hospital Laureano, além de trazer evidência para Kroeff. Assim, o diretor do SNC seguiu os passos de Antônio e Carmen Prudente.

Podemos considerar a atitude de Kroeff como uma reação à proposta de Prudente. Assim, o projeto paulista para o Brasil não passaria ileso pelo diretor do SNC, mesmo durante seu intercâmbio científico nos EUA.

Foi Sérgio de Barros Azevedo, diretor interino, quem recebeu a notícia sobre o “Plano Prudente” e escreveu para as autoridades governamentais, informando que o médico paulista “naturalmente não está em pleno conhecimento de nosso programa e de nossas atividades que continuam a ser exercidas intensivamente e com os maiores sacrifícios”, afinal, sua proposta “em linhas gerais coincide com o programa já estabelecido” (Azevedo, 3 mar. 1943). No entanto, esse programa não tinha sido entregue ao ministro Capanema, fato que obrigou Azevedo a se justificar no dia seguinte, afirmando que o programa já estava pronto, e não tinha sido entregue apenas porque esperava a deferência do diretor do serviço, ausente do país naquele momento. E ainda ressaltou: o projeto cuidou “do financiamento, por maneira mais prática que a sugerida pelo Dr. Antônio Prudente” (Azevedo, 4 mar. 1943).

Ao saber da situação, Kroeff escreveu uma carta detalhada ao ministro Capanema, na qual expressou desânimo e desilusão ao longo de todo o seu conteúdo. Ele criticou as considerações do médico paulista, apresentadas enquanto esteve ausente do país, por se chocarem com os interesses do próprio SNC:

Agora, porém, as notícias telefônicas, vindas do Brasil sobre os planos de Vossa Excelência, trouxeram-me pessoalmente a desilusão dos homens e a convicção de que é precário o nosso esforço ao bem público, quando depende de outrem. Permita confessar a Vossa Excelência que considero a reforma do Serviço Nacional do Câncer, feita durante a minha ausência, uma desconsideração ao seu diretor e uma ingratidão à minha pessoa, dados os serviços que tenho prestado aos doentes e ao meu país, através da medicina (Kroeff, 9 mar. 1943).

É curioso o fato de que o Instituto Arnaldo Vieira de Carvalho e o Hospital Humberto Primo, ambos em São Paulo, eram instituições consideradas “braços” do SNC; porém, a APCC não estava incluída. Apenas após o retorno de Kroeff – e para amenizar o mal-estar que se criou –, a Associação foi incorporada (Brasil, 19 out. 1943), o que compreendemos como um esforço do diretor do SNC para subordiná-la.

Para que a filiação se concretizasse, era necessário que a APCC enviasse seu estatuto para aprovação pelo presidente da República, no que Kroeff interferiu. Ao corresponder-se com o ministro Capanema, “pensando, entretanto, devia ser em caráter oficial” (Kroeff, s.d.), o diretor sugeriu algumas restrições:

No artigo 1º da ‘Associação Paulista de Combate ao Câncer’, onde se lê ‘com ação em todo o território nacional’, leia-se ‘subordinada ao Serviço Nacional de Câncer’.

No artigo 4º, letra b, onde se lê ‘Instituto de Câncer’, leia-se ‘Instituto Paulista de Câncer’ ou ‘Instituto Regional de São Paulo’.

... esse título Instituto de Câncer poderia futuramente acarretar colisões de nomenclatura e dualidade de atribuições, pois que ao Serviço Nacional de Câncer cumpre executar e controlar a luta contra o câncer em todo território nacional (Kroeff, s.d.).

Diante disso, podemos concluir que, ao contrário da França, cuja rivalidade entre cirurgiões e radiologistas resultou em disputas sobre concepções e terapias para o câncer (Pinell, 2004), as tensões entre os médicos de São Paulo e do Distrito Federal estavam relacionadas aos princípios ideológicos que embasavam seus projetos e, consequentemente, à questão de quem lideraria o combate ao câncer no país.

A conclusão do parágrafo anterior reforça, por um lado, o que foi observado por Andrade e Lana (2010) em uma pesquisa sobre a institucionalização do combate ao câncer no Brasil: existe uma disputa por primazia e protagonismo nas proposições de ações articuladas de combate à doença. Por outro lado, ao apresentar a comparação entre o Plano Prudente e o projeto de ação do SNC (Quadro 1), os historiadores entenderam que as lideranças em disputa partilhavam pressupostos semelhantes quanto à estruturação de políticas de saúde pública para o câncer. Todavia, essas semelhanças ocultaram as divergências ideológicas que nutriam as propostas.

**Quadro 1:** : Propostas da Rede Nacional contra o Câncer e o Plano Nacional de Combate ao Câncer

Rede Nacional Contra o Câncer	Plano Nacional de Combate ao Câncer
Construção de um Instituto Nacional de Câncer	Construção de um Instituto Central de Câncer
Divulgação de propaganda anticancerosa por todo o país	Intensa campanha de propaganda e de educação
Criação, em outros centros do país, de institutos regionais ligados às faculdades de medicina locais	Instalação de centros anticancerosos nas capitais, de preferência anexos aos hospitais gerais
Formação de técnicos e especialização de médicos ligados à Rede Nacional Contra o Câncer, bem como do público leigo	Educação de profissionais não médicos; educação dos médicos não especializados; e criação de cursos de aperfeiçoamento

Fonte: Andrade, Lana (2010, p.121).

Kroeff emergiu como figura proeminente na busca por estabelecer uma política centralizada no combate ao câncer, enquanto Prudente personificou a abordagem que colocava o aparato estatal sob a coordenação das organizações locais. Para Kroeff, a responsabilidade e o papel predominante na liderança dessa luta recaíam sobre o governo federal, enquanto, para Prudente, a União deveria desempenhar um papel subsidiário em relação às iniciativas privadas.

Na prática, no entanto, nenhum dos planos foi completamente efetivado. Ao longo da década de 1940, o SNC enfrentou dificuldades financeiras e permaneceu em situação frágil. Embora as ligas tenham ampliado sua atuação e obtido doações de particulares, elas receberam mais incentivos políticos e simbólicos da União do que subsídios concretos, o que só foi revertido em meados da década seguinte. Por outro lado, durante os anos de 1940, a APCC cresceu vertiginosamente e conseguiu arrecadar fundos para a construção do Hospital A.C. Camargo, inaugurado em 1953. Esse feito foi amplamente noticiado pelos jornais paulistas como “o início de uma nova era para a solução do câncer no Brasil” (Inaugurado..., 25 abr. 1953, p.11), alçando Prudente ao grupo dos principais nomes da cancerologia no país.

A década de 1950 foi um período marcante não apenas para a APCC ou para o cirurgião paulista, como também para São Paulo. Durante esse período, o estado foi apregoado, por meio de seus representantes, como exemplo de que nações tais como o Brasil tinham potencial para se integrar ao mundo industrializado. A capital paulista conquistou o título de maior cidade do país, superando o Rio de Janeiro em termos de população, e o estado abrigava mais indústrias que qualquer outra região da América Latina. Esse fenômeno impulsionou a realização de um enorme programa de comemorações, espetáculos e eventos culturais para celebrar o quarto centenário da fundação de São Paulo (Weinstein, 2015, p.231).

Nesse contexto, a chamada “medicina bandeirante” discursava a respeito das especialidades médicas empenhada na construção de uma São Paulo progressista e afinada com os novos tempos. Uma dessas especialidades era a própria cancerologia, que diplomou sua primeira turma no curso de residência em 1955, no próprio Hospital A.C. Camargo. Além disso, em janeiro de 1954, ao longo do segundo mandato de Getúlio Vargas (1951-1954), o cirurgião paulista foi nomeado diretor do SNC, sucedendo o seu idealizador e fundador, Mário Kroeff, que havia sido demitido por Miguel Couto Filho, então Ministro da Saúde. Assim, no decurso do quarto centenário de São Paulo, haveria a oportunidade para colocar em prática o projeto paulista estendido para todo o Brasil.

Sua primeira medida como diretor foi separar o SNC do Instituto de Câncer em sua parte física. O SNC foi deslocado para o 17º andar do Clube de Engenharia, um prédio no qual foram instalados vários departamentos do novo Ministério da Saúde, enquanto o instituto permaneceu nas dependências da Fundação Gaffrée e Guinle. Em seguida, iniciou a construção de três novos hospitais, localizados em Uberaba, Curitiba e Porto Alegre.

No plano da estratégia, Prudente instituiu o mês de maio como o período da campanha em todos os estados do país, com o propósito de angariar doações, seguindo a prática que já vinha adotando desde 1946 como líder da APCC, e, em julho, organizou o sexto Congresso Internacional do Câncer, realizado no Pavilhão das Indústrias do Parque Ibirapuera. Esse último evento reuniu cancerologistas de todo o mundo, com representantes de 52 países e quase mil congressistas, o que contribuiu significativamente para seu prestígio. Afinal, o congresso permitiu a inserção de diversos pesquisadores brasileiros em discussões globais sobre o tema.

Em suma, a escolha de Prudente para o cargo de diretor do SNC não foi aleatória, mas sim o resultado de disputas políticas que ocorreram desde a década anterior, culminando no ano simbólico de 1954. No entanto, sua passagem pela liderança do SNC foi muito breve. Após o suicídio do presidente Vargas, em agosto do mesmo ano, o ministro da Saúde foi destituído de sua função, e, junto com ele, seus nomeados. Assim, Prudente, que não completou sequer um ano na diretoria, foi substituído pelo cirurgião carioca Ugo Pinheiro Guimarães, que ocupou o cargo durante a gestão do presidente Café Filho.

Embora não tenhamos abordado o mandato de Guimarães, é interessante destacar que, mesmo alinhado a Mário Kroeff, o novo diretor foi obrigado a adotar, parcialmente, as medidas propostas por Prudente, alinhadas às discussões ocorridas no Congresso Internacional do Câncer. Dessa forma, ao escrever sobre a organização e execução da “luta anticancerosa no Brasil”, o novo diretor afirmou que:

A ação isolada, digamos, monopolizadora [do Estado sobre o problema do câncer], seria impraticável entre nós dada a extensão da campanha e a reconhecida impossibilidade material do governo em arcar com todo o ônus inevitável.

Um ou outro governo estadual considerou vantajoso constituir organismos locais próprios [referência a Prudente]. Entretanto, a tendência geral foi a de prestigiar a obra das organizações privadas que se fundaram. Esta mesma ação oficial estadual, quando existente, procura atuar em cooperação com a entidade privada filantrópica que funciona no Estado (citado em Teixeira, Fonseca, 2007, p.87).

O trecho sugere uma síntese das controvérsias que ocorreram na década de 1940, pois Guimarães reconhecia os desafios enfrentados no Brasil em relação às campanhas de combate ao câncer, devido à vastidão do país e às limitações financeiras do governo. Esse reconhecimento levou à autorização de entidades locais e governos estaduais para que tivessem autonomia na abordagem do problema do câncer em seus respectivos estados, desde que em cooperação com o SNC, estabelecendo uma parceria justificada entre os setores público e privado na luta contra os tumores malignos.

Destarte, ao longo da década de 1950, testemunhamos o surgimento de importantes hospitais especializados em câncer no país, localizados em Bahia, Rio Grande do Sul, Minas Gerais, Espírito Santo, Alagoas, Piauí, Paraná, Sergipe, Ceará e o já citado A.C. Camargo, em São Paulo. Essas instituições estavam vinculadas a ligas e associações de combate ao câncer que, por sua vez, estavam registradas no SNC e no Ministério da Saúde. Por fim, em 1957, foi inaugurado o Instituto Nacional do Câncer (Inca) no Rio de Janeiro, como parte da política de desenvolvimento nacional implementada pelo governo de Juscelino Kubitschek. O presidente conclamava o país a “agir contra as ‘doenças crônico-degenerativas’ ou as enfermidades do mundo desenvolvido, como o câncer” (Hochman, 2009, p.314-315).

Com isso, durante a década de 1950, as contingências históricas e políticas no Brasil impuseram limitações às iniciativas de Prudente como diretor do SNC. Apesar de ter sido afastado precocemente, sua proposta de uma abordagem descentralizada e em rede para o combate ao câncer encontrou eco entre seus sucessores, embora tenha sido motivada, em grande medida, pelas restrições materiais decorrentes da vastidão do país, e não por qualquer alinhamento ideológico ou eficácia de planejamento. Essa adoção parcial reflete a influência das ideias de Prudente no contexto do combate ao câncer no Brasil, bem como as limitações inerentes ao funcionamento do SNC.

## Considerações finais

Durante o recorte deste estudo, de 1934 a 1954, um plano de ação contra o avanço do câncer teve origem em São Paulo, que se desenvolveu e conquistou o apoio de diversas personalidades e da mídia, estendendo-se para além dos limites paulistas, logo, projetado como uma política anticâncer nacional. O médico Antônio Prudente foi a figura central desse movimento, e, não por acaso, o plano levou seu nome.

Na primeira parte deste artigo, apresentamos a fundação da APCC, que, longe de ser uma iniciativa isolada, legitimou diretrizes em consonância com outras ligas que surgiram no mesmo período. A novidade estava em seu formato consolidado por Prudente: primeiro, tanto ele quanto sua esposa, Carmen, utilizaram as representações do câncer para construir uma eficaz rede de relacionamentos e patrocínios; segundo, a APCC configurou-se como uma luta privada contra um mal público, em detrimento do próprio poder público – apesar de ter se aproveitado da estrutura sanitária de São Paulo para enfrentar e fundamentar o risco que o câncer oferecia; terceiro, esse processo foi marcado por um forte apelo ao sentimento de “paulistanidade”.

Na segunda parte do artigo, destacamos as estratégias do casal Prudente para conseguir cada vez mais donativos, assim como as contingências que os favoreceram nesse processo. Sublinhamos ainda a semelhança com a experiência francesa no período entre as guerras mundiais, cuja convocação para a luta contra o câncer assumiu uma qualidade patriótica. Em ambos os casos, o efeito foi uma mobilização veemente e passional, relevante para o sucesso das iniciativas. Em São Paulo, a materialização desse processo foi a criação do Hospital A.C. Camargo, em 1953, celebrado pela mídia paulista como “uma nova era para solução do câncer no Brasil” (Inaugurado..., 25 abr. 1953, p.11).

Na última parte, exploramos as disputas entre os cancerologistas de São Paulo, liderados por Prudente, e do Distrito Federal, chefiados por Kroeff. Enfatizamos que, até então, as pesquisas sobre o tema negligenciaram o substrato ideológico de tais disputas. Argumentamos que os pressupostos defendidos por Prudente se encaixam em um espectro ideológico liberal, característico de São Paulo durante o período em pauta, e foram materializados em um plano de ação denominado, informalmente, como Plano Prudente. Sua principal característica foi o estabelecimento de uma rede descentralizada de combate ao câncer que, ao diminuir os gastos da União, atribuía considerável autonomia aos governos estaduais, bem como às ligas e associações privadas, depositando excessiva confiança no patrocínio facultativo da população.

Embora Prudente tenha assumido a direção do SNC em 1954 e tido a oportunidade de implementar a Rede Nacional Contra o Câncer, sua saída precoce do cargo, devido ao suicídio de Vargas, impediu a completa realização de suas propostas. No entanto, sua influência perdurou entre seus sucessores, sendo motivada mais pelas necessidades e limitações materiais no atendimento das demandas nacionais que pelo alinhamento ideológico com a proposta de um plano paulista que buscava ser referência para todo o Brasil.

## NOTAS

1 A documentação utilizada neste estudo compreende correspondências trocadas entre os agentes históricos listados no texto, decretos oficiais de Estado, teses doutorais (que equivaleriam, hoje, aos trabalhos de conclusão de curso) apresentadas na Faculdade de Medicina de São Paulo, notícias divulgadas pelo jornal *O Estado de S. Paulo* e publicações (sejam livros ou artigos) do meio acadêmico médico. Coletamos as fontes no Centro de Pesquisa e Documentação de História Contemporânea do Brasil da Fundação Getúlio Vargas (https://www18.fgv.br/cpdoc/acervo/arquivo); acervo de obras raras da Faculdade de Medicina de São Paulo da Universidade de São Paulo; acervo Estadão (https://www.estadao.com.br/acervo/); e acervo da Biblioteca da Faculdade de Saúde Pública da Universidade de São Paulo.

2 A “paulistanidade” é um conceito que se originou na historiografia de São Paulo, cunhado por Alfredo Ellis Jr. em seu livro *Populações paulistas,* de 1934. Essa ideia descreve um conjunto de valores e mentalidades associados aos habitantes de São Paulo, com ênfase especial para a elite paulista, com seu núcleo mais evidente nos “respeitáveis coronéis” do interior do estado. O contexto da produção do livro de Ellis Jr. é marcado pela luta constitucionalista, e o autor não economizou elogios aos patriotas paulistas “que se fizeram voluntários do nosso exército constitucionalista” (Ellis Júnior, 1934, p.153). Posteriormente, o termo “paulistanidade” foi incorporado à historiografia de São Paulo de maneira crítica, denunciando seu conteúdo ufanista como uma ideologia que associava as virtudes políticas e culturais das “populações paulistas” ao crescimento econômico e demográfico que o estado experimentou na primeira metade do século XX. André Mota (2005), em sua tese de doutorado, analisou como essa ideologia se manifestou no campo médico, expondo os “tropeços da medicina bandeirante”. Messora (2017), por sua vez, demonstrou como esse ideário se expressava na busca de São Paulo pela vanguarda no tratamento contra o câncer, sobretudo entre 1920 e 1930, principalmente por se tratar de uma doença associada às “grandes civilizações”.

3 Em um contexto no qual as principais preocupações de saúde pública se relacionavam ao campo das endemias rurais, é coerente que o câncer, sem definições etiológicas, estivesse incluso sob o fisco da Inspetoria da Lepra, Doenças Venéreas e Câncer, braço do Departamento Nacional de Saúde Pública, criado em 1919. Em São Paulo, o Serviço Sanitário do Estado existia desde 1892, mas estava alinhado à Inspetoria na década de 1920, sobretudo, após a Reforma Carlos Chagas (1921), de modo que o câncer e os cancerosos eram vistoriados pelo Serviço Geral de Desinfecção e pela Secção de Estatística Demógrafa-Sanitária (Teixeira, Fonseca, 2007).

4 Na França, a partir de 1918, mulheres como a duquesa de Uzès ou a baronesa Mathilde mobilizaram inúmeros contatos e personalidades que colaboraram com trabalhos voluntários e doações generosas para os serviços de saúde franceses contra o câncer (Pinell, 2004). Já no Rio de Janeiro, ligas femininas surgiram a partir de 1953, mais associadas a atividades educativas direcionadas ao câncer que ao acúmulo de donativos para construção e manutenção de hospitais. Esse formato se justificava por dois motivos: primeiro, essas iniciativas se baseavam na American Cancer Society, que já reunia incontáveis mulheres para promoção de educação em saúde; segundo, porque as técnicas de prevenção contra os “cânceres femininos” expandiram-se no Brasil nesse período (Teixeira, Porto, Noronha, 2012, p.87).
